# Sinusoidal voltage protocols for rapid characterisation of ion channel kinetics

**DOI:** 10.1113/JP275733

**Published:** 2018-04-17

**Authors:** Kylie A. Beattie, Adam P. Hill, Rémi Bardenet, Yi Cui, Jamie I. Vandenberg, David J. Gavaghan, Teun P. de Boer, Gary R. Mirams

**Affiliations:** ^1^ Computational Biology, Department of Computer Science University of Oxford Oxford OX1 3QD UK; ^2^ Division of Applied Regulatory Science, Office of Clinical Pharmacology, Office of Translational Sciences, Center for Drug Evaluation and Research Food and Drug Administration Silver Spring MD USA; ^3^ Department of Molecular Cardiology and Biophysics Victor Chang Cardiac Research Institute Sydney NSW 2010 Australia; ^4^ St Vincent's Clinical School UNSW Sydney Darlinghurst NSW 2010 Australia; ^5^ CNRS & CRIStAL Université de Lille 59651 Villeneuve d'Ascq Lille France; ^6^ Safety Evaluation and Risk Management, Global Clinical Safety and Pharmacovigilance GlaxoSmithKline Uxbridge UB11 1BS UK; ^7^ Department of Medical Physiology, Division of Heart & Lungs University Medical Center Utrecht Utrecht The Netherlands; ^8^ Centre for Mathematical Medicine & Biology, School of Mathematical Sciences University of Nottingham Nottingham NG7 2RD UK

**Keywords:** potassium channel, mathematical model, ion current, voltage‐gated channels, voltage clamp, voltage gating

## Abstract

**Key points:**

- Ion current kinetics are commonly represented by current–voltage relationships, time constant–voltage relationships and subsequently mathematical models fitted to these. These experiments take substantial time, which means they are rarely performed in the same cell.
- Rather than traditional square‐wave voltage clamps, we fitted a model to the current evoked by a novel sum‐of‐sinusoids voltage clamp that was only 8 s long.
- Short protocols that can be performed multiple times within a single cell will offer many new opportunities to measure how ion current kinetics are affected by changing conditions.
- The new model predicts the current under traditional square‐wave protocols well, with better predictions of underlying currents than literature models. The current under a novel physiologically relevant series of action potential clamps is predicted extremely well.
- The short sinusoidal protocols allow a model to be fully fitted to individual cells, allowing us to examine cell–cell variability in current kinetics for the first time.

**Abstract:**

Understanding the roles of ion currents is crucial to predict the action of pharmaceuticals and mutations in different scenarios, and thereby to guide clinical interventions in the heart, brain and other electrophysiological systems. Our ability to predict how ion currents contribute to cellular electrophysiology is in turn critically dependent on our characterisation of ion channel kinetics – the voltage‐dependent rates of transition between open, closed and inactivated channel states. We present a new method for rapidly exploring and characterising ion channel kinetics, applying it to the hERG potassium channel as an example, with the aim of generating a quantitatively predictive representation of the ion current. We fitted a mathematical model to currents evoked by a novel 8 second sinusoidal voltage clamp in CHO cells overexpressing hERG1a. The model was then used to predict over 5 minutes of recordings in the same cell in response to further protocols: a series of traditional square step voltage clamps, and also a novel voltage clamp comprising a collection of physiologically relevant action potentials. We demonstrate that we can make predictive cell‐specific models that outperform the use of averaged data from a number of different cells, and thereby examine which changes in gating are responsible for cell–cell variability in current kinetics. Our technique allows rapid collection of consistent and high quality data, from single cells, and produces more predictive mathematical ion channel models than traditional approaches.

## Key points

- Ion current kinetics are commonly represented by current–voltage relationships, time constant–voltage relationships and subsequently mathematical models fitted to these. These experiments take substantial time, which means they are rarely performed in the same cell.
- Rather than traditional square‐wave voltage clamps, we fitted a model to the current evoked by a novel sum‐of‐sinusoids voltage clamp that was only 8 s long.
- Short protocols that can be performed multiple times within a single cell will offer many new opportunities to measure how ion current kinetics are affected by changing conditions.
- The new model predicts the current under traditional square‐wave protocols well, with better predictions of underlying currents than literature models. The current under a novel physiologically relevant series of action potential clamps is predicted extremely well.
- The short sinusoidal protocols allow a model to be fully fitted to individual cells, allowing us to examine cell–cell variability in current kinetics for the first time.

## Introduction

Mathematical models of ion channels are a quantitative expression of our understanding of ion channel kinetics: they express the probability of channels existing in different conformational states (typically, closed, open and inactivated) and the rates of transition between these states (Bett *et al*. 2011; Vandenberg *et al*. 2012). Parameterising/calibrating a mathematical model of an ion current is a concise way to characterise ion channel kinetics, to capture our understanding in a quantitative framework, and to communicate this knowledge to others. There have been some notable advances in deriving mathematical models for ion channel behaviour (Balser *et al*. 1990; Cannon & D'Alessandro, 2006; Siekmann *et al*. 2011, 2012; Loewe *et al*. 2015), with some stressing the need for validation/testing of the model using data from the same cell (Tomaiuolo *et al*. 2012). In this paper we present a new approach for characterising ion channel kinetics, using novel short protocols and parameter inference techniques to construct an ion channel model.

**Figure 1:**
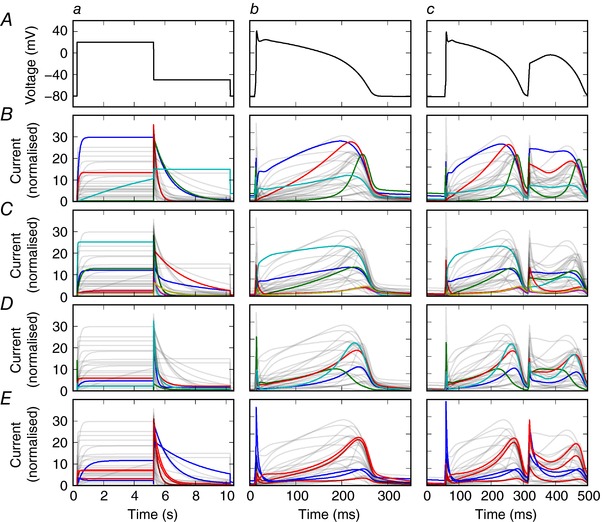
Predicted currents from literature models of *I*_Kr_ Each column shows simulated current predictions from 29 *I*_Kr_ literature models in response to the different voltage clamp protocols shown in the top row. *A*, voltage clamps: (*a*) a voltage step; (*b*) an action potential; and (*c*) an action potential displaying pathological properties. Each of the panels below features all 29 current predictions in faint grey, to aid comparison between plots. *B*, the four models for canine ventricle at physiological temperature. *C*, the six models for human ventricle at physiological temperature. *D*, the four models for rabbit sino‐atrial node at physiological temperature. *E*, the five models for hERG1a expression systems: at room temperature in blue and physiological temperature in red. Currents are normalised such that the maximal conductance is equal to 1; i.e. we plot the open probability multiplied by the driving voltage (all model references and structures are listed in Supporting information, Appendix A, Table A1). All models have been simulated with their original published parameters, with the same reversal potential of −88.4 mV.

The *KCNH2* gene (also known as *hERG*) has been shown to encode the primary subunit of the voltage‐gated ion channel Kv11.1 that carries the rapid delayed rectifier potassium current (*I*_Kr_) (Sanguinetti *et al*. 1995; Trudeau *et al*. 1995). In this article we focus on mathematical modelling of hERG channel kinetics, demonstrating our approach by constructing an improved model of this ion current. hERG plays important roles in the brain (Babcock & Li, 2013), gastrointestinal tract (Farrelly *et al*. 2003), uterine contractions (Parkington *et al*. 2014), cell proliferation and apoptosis (Jehle *et al*. 2011), and cancer progression (Lastraioli *et al*. 2015), but *I*_Kr_ is best known as a repolarising cardiac ion current. The channel is susceptible to binding and blockade by pharmaceutical compounds, which is strongly linked to many cases of drug‐induced pro‐arrhythmic risk (Redfern *et al*. 2003; Pollard *et al*. 2010). Mathematical modelling of cardiac electrophysiology, including *I*_Kr_, forms a core part of a new proposal for routine *in vitro* and *in silico* safety assessment to replace a human clinical drug safety study (Sager *et al*. 2014; Fermini *et al*. 2016). A wide range of different mathematical models have been proposed to describe *I*_Kr_ (literature models are listed in online Supporting information, Appendix, Table A1). Figure 1 shows predicted *I*_Kr_ under three different voltage clamps for 29 literature models. These models were developed to describe different species, cell types, temperatures and isoforms, so variation is expected. In Fig. 1 *B*–*E* each row highlights models developed to represent the same species, cell type and temperature; even models for the same conditions provide highly variable predictions.

The first models of ion channel kinetics were proposed by Hodgkin and Huxley (1952), and relatively little has changed in the methods used for construction of mathematical models of ion channel gating since the original seminal work in this journal in 1952. Their (now traditional) approach was to fit peak currents and time constants of current activation/decay after clamping to fixed voltages, to assemble current–voltage (*I–V*) and time constant–voltage (τ–*V*) curves, and to describe these curves with interpolating functions.

Condensed voltage clamp step protocols have been suggested as the basis of optimised experiments that provide information about ion channel kinetics faster than experiments to construct *I–V* curves (Hobbs & Hooper, 2008; Fink & Noble, 2009), and optimised current and square step voltage clamps have been used to optimise the fitting of maximal conductances in action potential models (Groenendaal *et al*. 2015). Single sinusoid voltage clamps have been previously been explored for choosing between possible Shaker channel models that were parameterised using traditional square step voltage clamps (Kargol *et al*. 2004). Wavelet‐based voltage protocols have also been suggested for examining sodium channel dynamics (Hosein‐Sooklal & Kargol, 2002). The study by Kargol (2013) features excellent insight into the problem of models behaving similarly under traditional clamps but differently under optimised information‐rich protocols. In that paper, these wavelet‐based protocols were designed and used to select between Shaker potassium channel models.

In this study, we extend these ideas and propose an 8 s sum‐of‐sinusoids‐based voltage clamp, designed to both explore and fully characterise the kinetics of the hERG potassium channel. We use this new protocol to record currents from Chinese hamster ovary (CHO) cells that were over‐expressing hERG1a. These recordings were then used to parameterise a mathematical model which became our characterisation of the ion current. We then evaluated the model by predicting the response to both standard square step voltage‐clamp protocols and, perhaps more importantly, physiologically relevant action potential voltage clamps, using these data (which were independent of the recordings used to fit the model) to perform an extremely thorough validation for the model of ion channel kinetics. Our approach uses a substantially shorter experimental recording to construct the model than the usual approach, which is based on time constants and peak currents from a long series of square step voltage‐clamp protocols. As a consequence of the high information content of the short protocol, we are able to generate cell‐specific models that advance our understanding of variability of ion currents between cells. Our methodology will be applicable to many ion channels, both in the heart and other electrophysiological systems.

## Methods

### Experimental methods

**Figure 2:**
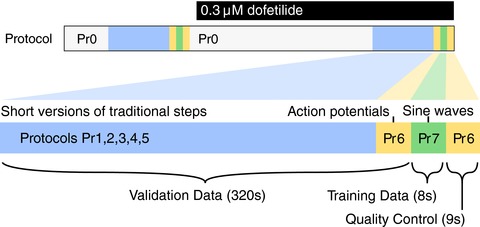
Schematic diagram of the experimental procedure used in this study (not to scale) A simple activation step protocol is repeated in the sections marked Pr0, before moving on to the highlighted section (below) where data used in the study were recorded. The recording protocols Pr1–7 are performed twice, once before dofetilide addition, and once after, with the hERG current isolated by subtraction. For full details of the protocols, refer to Supporting information, Appendix B1.4. In each cell we recorded the following: a series of conventional voltage‐step protocols designed to explore activation (Pr1–3), inactivation (Pr4) and deactivation (Pr5); a new protocol composed of a series of action potential clamps (Pr6 – formed of simulated action potentials from different mathematical models to represent diverse species and pacing frequencies in both healthy and repolarisation‐failure conditions); and our new 8 s sinusoidal voltage protocol (Pr7, shown in Fig. 3). These protocols are all performed in a single experiment using a single cell, and the process can be repeated in different cells. A mathematical model is then fitted/calibrated to solely the current provoked by the sinusoidal protocol, and this model then represents a full characterisation of *I*_Kr_ in each particular cell. The characterisation is then tested for accuracy by using the fitted mathematical model to predict the results of all the other voltage clamp protocols performed in that cell. Full details of all protocols are given in Supporting information, Appendix B1.4. [Color figure can be viewed at http://wileyonlinelibrary.com]

We performed whole‐cell patch‐clamp voltage‐clamp experiments using CHO cells stably expressing hERG1a (Kv11.1) at room temperature. Full details including cell culture, solutions, and equipment settings can be found in online Supporting information, Appendix B. Figure 2 provides an overview of the experimental approach, denoting the sequence of voltage clamp protocols we performed as Protocols (Pr) 0–7.

In all protocols, the holding potential was initially −80 mV before applying a 50 ms leak step to −120 mV before returning to −80 mV, with this step being used to estimate leak current (as described below in the section ‘Leak corrections’). A voltage step to −120 mV at the end of all the protocols ensures that channels close quickly, reducing the time needed between protocols to regain a steady closed state.

#### Protocols 0 to 5: square step clamps

Pr0 was a simple repeated activation pulse designed to open the channel to visually test that the recordings were stable and to allow dofetilide binding, considered open state dependent, to occur (see the section ‘Dofetilide subtraction’ below). This current was not recorded or used in the subsequent analysis (hence the name ‘Protocol 0’).

Pr1–5 were adaptations of ‘traditional’ square step voltage clamps used in previous studies to examine activation (Pr1–3), inactivation (Pr4) and deactivation (Pr5). Details of the protocol voltages and timings can be found in Supporting information, Appendix B1.4. The ‘adaptation’ is that Pr1–5 are shorter than those previously used to calibrate mathematical models (as fewer test voltages/timings are used), so that it is possible to perform them all in a single cell, with and without dofetilide subtraction. A ‘traditional approach’ would take longer than the experiments performed here, generally requiring multiple cells.

#### Protocol 6: action potential clamp

Pr6 was formed by combining a series of different simulated action potentials from the Cardiac Electrophysiology Web Lab (Cooper *et al*. 2016). The range of models we used for the simulations encompassed different cell types, species and pacing rates. We also added some simulated action potentials where early or delayed after‐depolarisations had been induced, to test *I*_Kr_ behaviour in pro‐arrhythmic or pathological settings. The action potentials were shifted slightly so that their resting potentials were exactly −80 mV (see Supplementary code for full details and code to reproduce this protocol).

#### Protocol 7: sinusoidal clamp

The protocol used to characterise the current and train the model was a voltage clamp comprising simple steps and a main sinusoidal section that was in the form of a sum of three sine waves of different amplitudes and frequencies, designed to rapidly explore hERG channel kinetics. The underlying rationale was to force the protocol to ‘sweep’ both the time and voltage dependence of the current gating over physiological voltage ranges.

The start of the protocol took the form of a leak step followed by a simple activation step that was similar to Pr0. This activation step was included to improve the identifiability of the maximal conductance parameter (as described in Supporting information, Appendix B2.2) after preliminary experiments suggested this might improve what is known as ‘parameter identifiability’ (to pin down possible values of the parameter more accurately, and prevent other kinetic parameters compensating for an inaccurate conductance value).

The main sinusoidal portion of the protocol took the form of a sum of three sine waves:

(1) $$\begin{array}{ccc} V(t) & = & -30+A_{1}\sin\left(\omega_{1}\left(t-t_{0}\right)\right)\\ & & +\;A_{2}\sin\left(\omega_{2}\left(t-t_{0}\right)\right)\\ & & +\;A_{3}\sin\left(\omega_{3}\left(t-t_{0}\right)\right), \end{array}$$

where *A*_1_ = 54 mV, *A*_2_ = 26 mV, *A*_3_ = 10 mV, ω_1_ = 0.007 ms^−1^, ω_2_ = 0.037 ms^−1^ and ω_3_ = 0.19 ms^−1^, and *t* is time in milliseconds. In terms of frequencies, existing models and *I*_Kr_ recordings included characteristic time scales of the order of 10 ms to 1 s (Wang *et al*. 1997; Zhou *et al*. 1998). Therefore, we designed the sinusoidal protocol's three frequencies to probe channel kinetics across all these orders of magnitude (10 ms, 100 ms and 1 s time scales). We selected frequencies that were co‐prime rather than exactly multiples of 10: ω_1_ to ω_3_ are ordered slow to fast and correspond approximately to sine waves of period 900, 170 and 33 ms, respectively. The aim was that the three distinct frequencies should not become ‘in phase’: the protocol never repeats patterns that the cell has experienced before (ensuring new information is supplied throughout). The offset *t*_0_ is 2500 ms as explained in Supporting information, Appendix B1.4. If one were to study other ion channels, these frequencies may need adjustment to examine relevant time scales.

To decide the amplitudes, the oscillations were centred around −30 mV so that a physiological range is explored (−120 < *V* < 60 mV). The amplitudes of the sine waves were selected to keep the protocol within this range (*A*_1_ + *A*_2_ + *A*_3_ = 90 mV) and to ensure that *A*_1_ > *A*_2_ > *A*_3_ so that the fastest time scale had the smallest oscillations (to avoid the faster gating processes masking the voltage dependence of slower ones). A key step in settling on this particular protocol was its performance in synthetic data studies. In these studies we simulated *I*_Kr_ with different sets of given parameters, then attempted to recover these parameters blindly – using just the generated current trace with added noise, as illustrated in Supporting information, Appendix C2 (we also show this for an *I*_Ks_ model with the same protocol in Supporting information, Appendix G).

**Figure 3:**
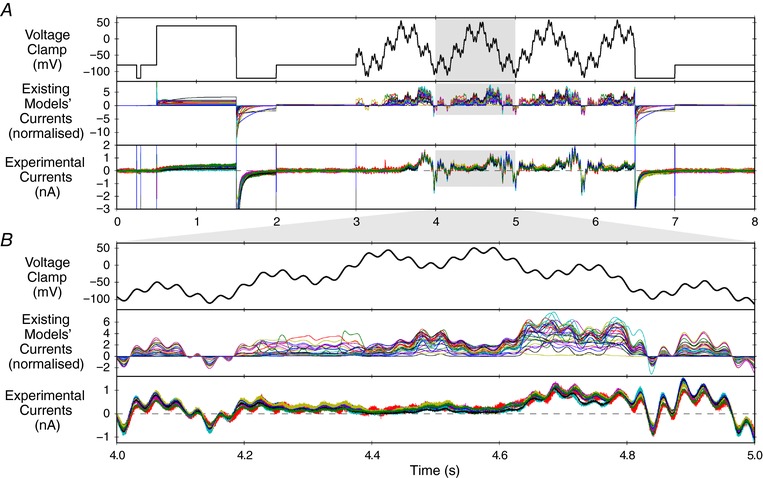
The sinusoidal protocol and example recordings *A*, top row, the full sinusoidal voltage protocol (Pr7). Middle row, simulations of expected behaviour in response to this protocol from existing *I*_Kr_ and hERG models, normalised by scaling the conductance value for each model to minimise the absolute difference between each trace and a reference trace. For calculation of the reversal potential, a temperature of 21.5°C was used to match the mean experimental conditions. Bottom row, raw data (following leak and dofetilide subtraction) from experimental repeats at room temperature from 9 cells. Experimental traces have been scaled, to remove the effect of different maximal conductances, by a factor chosen to minimise the absolute differences between each trace and a reference experimental trace (that with the peak current during the sinusoidal portion of Pr7). *B*, an enlargement of the highlighted sections of panel *A*. While there is some variation between cells in the experimental results, they are much more consistent than the predictions from the different models. [Color figure can be viewed at http://wileyonlinelibrary.com]

The sinusoidal protocol was of only 8 s duration, which enables efficient data collection, with training and validation data collected from the same cell. Figure 3 shows the novel sinusoidal protocol Pr7, the simulated predicted currents from existing models and the currents that were recorded experimentally. The new protocol provokes an even wider array of different behaviours from the existing literature *I*_Kr_ models (middle panels in Fig. 3) than the existing voltage step or action potential clamps (Fig. 1), even among models constructed in/for similar conditions/species.

### Leak corrections

We used the leak step from −80 to −120 mV in order to leak‐correct the experimental data, according to:

(2) I corrected =I raw −VR leak .

We identified the most appropriate *R*_leak_ value to minimise the difference between the mean current value during the leak step (to −120 mV) compared to the mean value at a holding potential of −80 mV, whilst ensuring that the trace was not overcorrected (which would result in negative currents during the initial stages of activation). We manually selected leak resistances to correct the current evoked by the sinusoidal protocol in both vehicle and dofetilide conditions. We then applied this leak resistance to the remaining protocols performed in the same condition on each cell. The mean current during the −80 mV step was calculated from 200 ms of the −80 mV holding period before the −120 mV leak step (not including the capacitive spike at the point at which the step occurs). The baseline current at a holding potential of −80 mV was then adjusted back to 0 nA with an additional constant additive current if required.

### Dofetilide subtraction

In preliminary work, we observed that our sinusoidal protocols could elicit endogenous voltage‐dependent background currents within expression‐system cells. We observed that the levels of endogenous currents the protocols elicited varied from cell to cell. These currents could adversely affect the predictive ability of the resulting mathematical models, as the fitting process attempted to create a model that described both the endogenous and *I*_Kr_ components of the recorded currents. To overcome this technical issue, we made a number of alterations to our pilot experiments. Firstly, we constrained the design of the sinusoidal protocol, as discussed above, so that only voltages within a physiological range of −120 to +60 mV were explored, as endogenous currents were much more prominent at voltages above +60 mV that we explored in pilot studies. Secondly, we changed to using CHO cells in this study, rather than the HEK cells we used in pilot studies, as CHO cells generally had lower endogenous currents. Thirdly, we recorded the full set of voltage protocols (Pr1–7) twice: once in dimethyl sulfoxide (DMSO) vehicle conditions and once following the addition of 0.3 μm dofetilide, as shown in Fig. 2. Dofetilide was first dissolved in DMSO before being added to the bath solution to produce the required concentration. The required dose of dofetilide was obtained by serial dilution. We chose to use 0.3 μm because the dofetilide hERG IC_50_ value is <10 nm, which, assuming a Hill coefficient of 1, should correspond to >97% conductance block of *I*_Kr_ at 0.3 μm dofetilide. We avoided higher concentrations as dofetilide has other known voltage‐dependent ion channel targets whose IC_50_ values are in the tens to hundreds of micromolar range (Mirams *et al*. 2011). Between the two recordings we allowed the dofetilide‐induced current block to reach equilibrium (under Pr0). We then subtracted the currents that remained in the presence of dofetilide from those recorded in the presence of vehicle to remove any contribution of endogenous currents (and to produce what we refer to as ‘dofetilide subtracted’ data). Prior to performing this subtraction, we first leak‐subtracted both the vehicle and dofetilide recordings individually, as described above. It may not always be necessary for dofetilide subtraction to be performed on CHO cells, as endogenous voltage‐dependent currents can be very low, and leak subtraction may suffice (see Supporting information, Appendix B1.6). But we applied the dofetilide subtraction method nonetheless to generate a gold‐standard dataset for this study.

### Mathematical model

**Figure 4:**
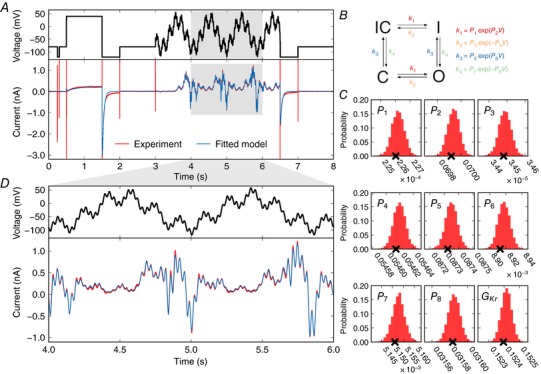
Model calibration *A*, top, the entire 8 s training protocol; bottom, an experimental recording with the fitted model simulation overlaid (portion of the sinusoid enlarged in panel *D*). This simulation uses the maximum posterior density parameter set, denoted with crosses in panel *C*. *B*, the model structure in Markov state diagram format; note that the symmetric transition rates mean this is equivalent to a Hodgkin–Huxley‐style model with two independent gates. Parameter values *P*_1_–*P*_8_ define voltage (*V*)‐dependent transitions (*k*) between conformational states. *C*, posterior distribution of single‐cell‐derived model parameters. Probability density distributions are shown for each parameter after fitting to the experimental data shown in panel *A*. The parameter numbering corresponds to that shown in panel *B*. Crosses indicate the parameter set with the maximum posterior density. The standard deviation of each of these distributions is less than 0.2% of the maximum posterior density value. *D*, an enlargement of the highlighted region of panel *A*.

Whilst our model is equivalent to a two gate Hodgkin–Huxley formulation, we use a Markov model description in practice (simply to generalise the computational code for other model structures; the relationship between equivalent Markov and Hodgkin–Huxley models is explained in Keener and Sneyd (2009), vol. 1, p150). The system of ordinary differential equations underlying the mathematical model structure shown in Fig. 4 *B* is then:

(3) $$\;\frac{d[C]}{dt}=\;-\left(k_{1}+k_{3}\right)\left[C\right]+k_{2}\left[O\right]+k_{4}\left[\mathrm{IC}\right],$$

(4) $$\;\frac{d[O]}{dt}=\;-\left(k_{2}+k_{3}\right)\left[O\right]+k_{1}\left[C\right]+k_{4}\left[I\right],$$

(5) $$\;\frac{d[I]}{dt}=\;-\left(k_{2}+k_{4}\right)\left[I\right]+k_{3}\left[O\right]+k_{1}\left[\mathrm{IC}\right],$$

where the fourth state is constrained by probabilities of state occupancies summing to one

(6) $$\left[\mathrm{IC}\right]=\;1-\left(\left[C]+[O]+[I\right]\right).$$

The eight parameters *P*_1_⋯*P*_8_ determine the transition rates *k*_1_⋯*k*_4_ according to the exponential voltage dependence relationships shown in Fig. 4 *B*. The current, *I*_Kr_, was modelled with a standard Ohmic expression:

(7) I Kr =G Kr [O](V−EK),

where *G_Kr_* is the maximal conductance, *E*_K_ is the Nernst potential for potassium ions, and [O] is the open probability, given by the solution to the system of equations above. *E*_K_ was not inferred, but was calculated directly from the ratio of ion concentrations on each side of the cell membrane using the Nernst equation:

(8) $$E_{K}=\frac{RT}{zF}\;\ln\frac{{\left[K\right]}_{\mathrm{out}}}{{\left[K\right]}_{\mathrm{in}}}.$$

where *R* is the ideal gas constant, *T* is the temperature, *F* is the Faraday constant, *z* is the valency of the ions (in this case 1), and [K] represents the concentration of potassium ions. Note that this expression has a temperature dependence, and the temperature of the bath was recorded for each cell and used in relevant simulations. All simulations were performed in MATLAB (The MathWorks Inc., Natick, MA, USA). Mex functions were used to define the equations and simulate by using CVODE (Hindmarsh *et al*. 2005) to solve the systems of differential equations, with both absolute and relative tolerances set to 10^−8^. Code is available to download as described under ‘Additional information, Data deposition’.

### Parameter inference

We used a global minimisation algorithm (Hansen *et al*. 2003) followed by a custom‐written Bayesian inference method. Parameters were estimated using a Monte Carlo based inference scheme, in this case using an approach similar to that described in Johnstone *et al*. (2016). In Supporting information, Appendix B2 we give details of how (1) a likelihood is assigned to any candidate parameter set; (2) maximising the likelihood using a global optimisation scheme gives a ‘best fit’ parameter set; and (3) uniform prior distributions are assigned to the kinetic parameters; and (4) we start a Markov chain Bayesian inference scheme from the estimated global optimum to generate a posterior probability distribution. The benefits of this scheme are that we explore the ‘parameter space’ widely and build up a probability distribution (probability of parameters generating the experimental results we observed) across the whole parameter space, thereby characterising any uncertainty in the ‘best fit’ parameter set. This posterior distribution allows us to check that we are constraining each parameter's value with the information in the experiment and are not experiencing problems with identifiability of parameters (Siekmann *et al*. 2012).

### Note on normalisation

Where existing literature model simulations were plotted alongside experimental traces, or one experimental trace was compared with another, we first had to normalise to account for differences in conductance values. This was achieved by selecting a scaling factor for the conductance value for each model simulation (or experimental trace) that minimised the square difference between each trace and a reference experimental trace. For literature models the reference trace was the experimental current from the action potential clamp Pr6. Note this provides a best‐case fit to Pr6 for all of the literature models, removing the possibility that some models open ‘half as much’ because they have ‘twice the conductance’. For the new model, no scaling was applied and conductance was directly fitted to the experimental current from the sinusoidal protocol (along with other parameters).

## Results

### Model calibration

We calibrated a mathematical model using only data recorded under the sinusoidal protocol (Pr7). The Hodgkin–Huxley‐style structure of the model we used, and its corresponding model parameters, can be seen in Fig. 4 *B*. We independently fitted this model to each of the experimental current traces shown in Fig. 3. For each cell, we obtain a probability distribution of estimates for each parameter that captures any observational uncertainty in the parameter values (Pathmanathan & Gray, 2013; Mirams *et al*. 2016).

The result of the fitting procedure for one cell is shown in Fig. 4. The parameter set with maximum posterior density is shown in Fig. 4 *A*, demonstrating an excellent fit between experimental and simulated data. The resulting posterior probability density for the parameters obtained from this Bayesian inference approach is projected across each parameter in Fig. 4 *C*. We also tested that our approach is theoretically appropriate for inferring all parameters by using synthetic data studies, as described in Supporting information, Appendix C. The plausible parameter space is very narrow: if multiple parameter set samples are taken from the distribution shown in Fig. 4 *C*, the resulting simulated current traces are indistinguishable to the eye. To quantify this, taking 1000 samples we found that the 95% credible intervals for the simulated currents were always within at most either 3.47% or, in absolute terms, 0.0043 nA of the simulated current given by the maximum posterior density parameter set.

The results presented in Fig. 4 are from a single cell with a good quality recording and a high signal:noise ratio (this choice of cell, and other cells’ predictions, are discussed later). We fitted models on a cell‐specific basis, and then also use averaged experimental data to create a single ‘averaged’ model as described in Supporting information, Appendix F. We compare these approaches below. We provide all parameter values with the maximum posterior density for all models in Supporting information, Appendix Table F11.

### Validation predictions

**Figure 5:**
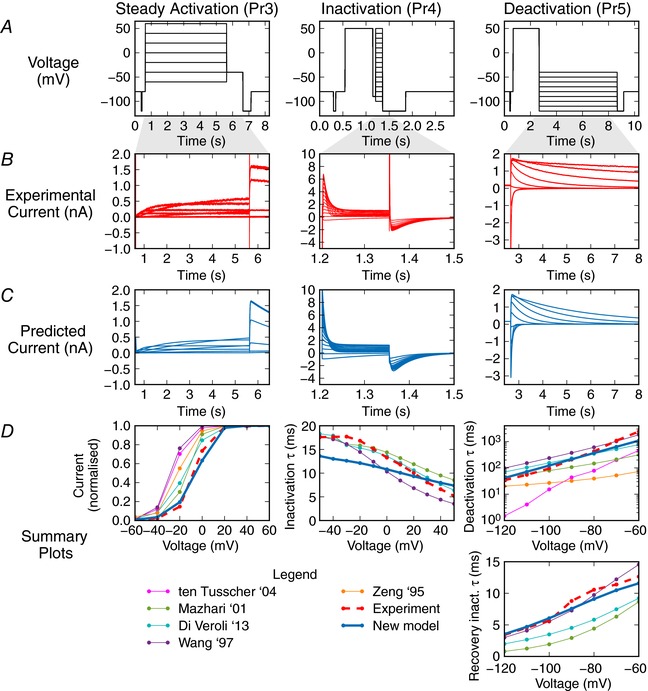
Validation predictions – currents in response to traditional voltage step protocols Each column of graphs corresponds to a validation step protocol: those commonly used to study steady state activation, inactivation and deactivation (Pr3, Pr4 and Pr5 in Fig. 3), respectively. *A*, the voltage protocols. *B*, experimental current traces. *C*, model response – all are predictions using the maximum posterior density parameter set indicated in Fig. 4 *C* calibrated to just the sinusoidal protocol. *D*, summary curves, either current–voltage (*I–V*) or time constant–voltage (τ–*V*) relationships. These plots summarise the results in the relevant column. The model prediction is shown in blue bold throughout, and the experimental recording with a dashed red line. The deactivation time constant plotted here is a weighted tau, described in Supporting information, Appendix B1.7. Note that some literature model predictions are missing from the summary plots as we were either unable to fit exponential curves to ‘flat’ simulation output reliably, or the exponential decay occurred in the opposite direction to experimental traces, and we considered the comparison unwarranted.

**Figure 6:**
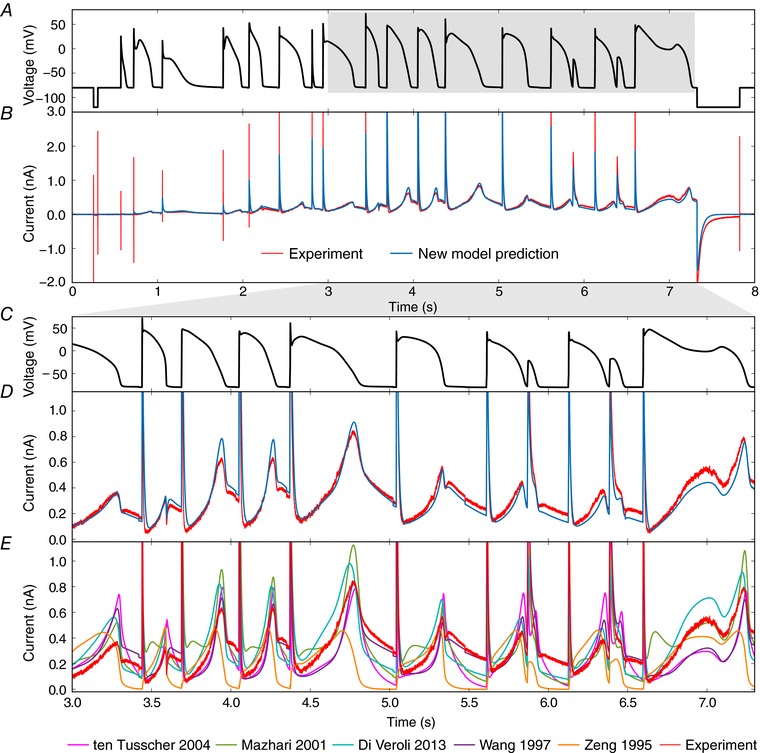
Validation prediction – the current in response to the action potential protocol *A*, the voltage clamp protocol. *B*, a comparison of the experimental recording (red) and new model prediction (blue). *C* and *D*, enlargements of the highlighted regions of panels *A* and *B*. *E*, the same view of the experimental data in panel *D*, but here compared with predictions from literature *I*_Kr_ models. Conductance, *G*_Kr_, is scaled for each of the literature models to give the least square difference between their prediction and these experimental data, i.e. we display a best‐case scaling for each of these models. A quantification of the error in our model prediction *versus* these literature models is given in Supporting information, Appendix Table D6: the performance shown in panels *D* and *E* holds for the whole trace, so the mean error in predicted current across the whole protocol is between 69% and 264% larger for the literature models’ predictions than for our sine‐wave fitted model.

Having trained our model to 8 s of experimental data from the sinusoidal protocol Pr7, we now tested its ability to predict more than 5 min of independent experimental behaviour. We predicted the current in response to traditional voltage‐step protocols, Pr1–5 (adapted from those previously used in the literature; Bett *et al*. 2011), and also to a novel physiologically inspired voltage clamp protocol comprising multiple action potentials (Pr6). All recordings shown in Figs 4, 5, 6 are from the same cell, using the experimental procedure shown in Fig. 2. To make the predictions for Pr1–6, we performed simulations using the parameter set with the maximum posterior density in the fit to the sinusoidal protocol (Pr7). As with the calibration protocol, all the predictions discussed below are indistinguishable by eye from the result of taking multiple samples from the distributions in Fig. 4 *C* and plotting a prediction for each of these parameter sets.

Figure 5 shows traditional voltage step protocols, experimental recordings and the simulated predictions from the model. It also shows some of the most commonly plotted summary curves for experimental data under these protocols, together with predicted summary curves from our model. We compare these results with the summary curve predictions from a sample of widely used literature models. We chose models for hERG1a expression systems at room temperature (Wang *et al*. 1997; Di Veroli *et al*. 2013) and physiological temperature (Mazhari *et al*. 2001); and also models with the same Hodgkin–Huxley structure as ours (Zeng *et al*. 1995; Ten Tusscher *et al*. 2004) albeit for physiological temperatures, as these are most directly comparable (methods used to derive summary plots are given in Supporting information, Appendix B1.7 with some additional summary curves for Pr1, 2 and 4 in Appendix E). We could predict a wide range of current behaviour in response to the standard voltage‐step protocols, without having used any of this information to fit the model.

There are a number of points to draw attention to in Fig. 5. Firstly, most of the *I*–*V* relationships and τ–*V* relationships we predicted in response to the traditional voltage‐step protocols were closer to the experimental data than similar model–experiment comparisons in the literature (even when existing literature models, with more parameters, were fitted to such data). Secondly, there were some weaknesses to the new model – particularly in predictions of the Pr4 summary plot of τ of inactivation against voltage, where we predicted a time constant that was approximately 4 ms too fast at −40 mV. Yet, it is worth noting that this may be the best fit that is possible with a Hodgkin–Huxley‐style model: the Ten Tusscher and Zeng models predict time courses that are so different it is difficult to fit comparable time constants.

The current time course for Pr4 is actually predicted more accurately than any of the other models shown here (see Supporting information, Appendix Table D6) despite the τ–*V* relationship being less accurate; in agreement with this, other summary *I–V* curves of Pr4 are predicted more accurately by the new model (see Supporting information, Appendix Figs E9 and E10).

Figure 6 shows the model prediction of the currents invoked in response to the physiologically inspired action potential protocol Pr6, compared with the experimental recording (as shown in Fig. 2, we used the first repeat of Pr6 for validation purposes, and the second as a quality control measure).

Replicating behaviour under action potentials is perhaps the most important requirement for a hERG channel model for use in physiological or pharmacological studies. The model is able to predict the response to all of the complex action potential protocol extremely well, and much better than existing models (even though we have scaled all the literature models’ maximal conductances (*G*_Kr_) to fit this trace as well as possible in Fig. 6).

We provide a quantitative comparison of predicted current traces for our model and each of the literature models for Pr3–7 in Supporting information, Appendix Table D6. In each case, the worst‐performing literature model is a Hodgkin–Huxley‐style model. Yet our simple model, with the same structure, is able to provide significantly better predictions than even the Markov‐type models, which are usually considered to be better representations of hERG kinetics (Bett *et al*. 2011). Our methodology has resulted in a simple and highly predictive mathematical model, able to describe a wide range of physiologically relevant behaviour.

#### Cell‐specific validation

**Figure 7:**
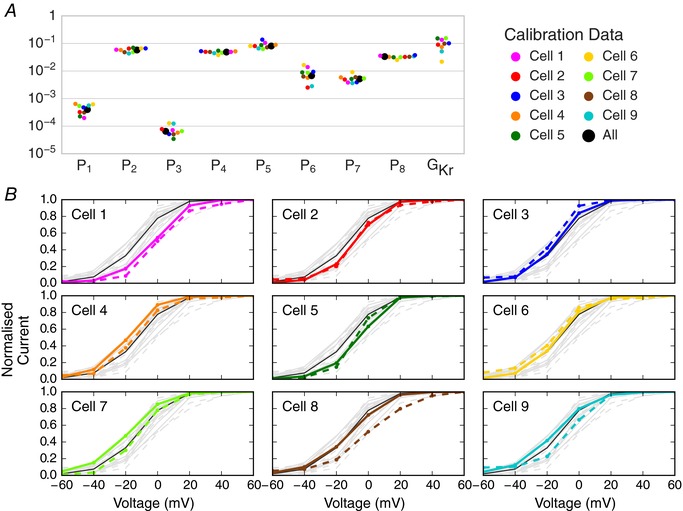
Cell‐specific model parameters, and comparison of their predictions with cell‐specific experimental results *A*, plot of parameters (maximum posterior density values) for nine cells obtained from training the model to the sinusoidal voltage protocol recorded on nine different cells, together with parameters calibrated to average data (N.B. not the average of the cell‐specific parameters). The full set of parameter values are shown in Supporting information, Appendix Table F11 and the distributions for each parameter shown in Appendix Fig. F11. *B*, comparison of cell‐specific model predictions to cell‐specific experimental recordings for the steady‐state peak current *I–V* curves from Pr3. Each plot represents a different cell; model predictions are depicted by a bold coloured line and dashed lines show values derived from the experimental data. The black lines (same on each plot) represent the prediction from the model calibrated to averaged sinusoidal data (all of the cells’ data). Each subplot contains all of the other cells’ recordings and predictions in light grey in the background to aid comparison and show the spread that we observed.

Figure 7 *A* shows the maximum posterior density parameter values when repeating the above approach using data from nine different cells. The clustered parameter values demonstrate that parameters derived from different cells take similar values, giving us confidence that the procedure is reproducible and biophysically meaningful. There is more cell‐to‐cell variability in some parameters than others, which may be related to variability in the underlying physiological processes that they represent, supporting the value, and perhaps necessity, of a cell‐specific approach. We also acknowledge that some parameters may be more or less sensitive to variability in experimental conditions such as temperature, residual background/endogenous currents, and imperfect dofetilide and/or leak subtraction.

We ordered the cells in Fig. 7 based on the lowest to highest difference in leak resistance between the vehicle and dofetilide recordings of Pr7. This ordering gives a measure of recording stability and is intended to be a surrogate for data quality. The cell presented above, in Figs 4, 5, 6, corresponds to Cell 5 of nine under this ranking, so we obtained very good predictions even with our ‘median’ quality data. Cell‐specific predictions of the *I–V* relationship for the peak steady‐state activation current for each cell‐specific model is shown in Fig. 7 *B*. While we focused on Cell 5 in Results, Cells 1–4 also produced excellent cell‐specific predictions (similar comparisons for other summary plots are in Supporting information, Appendix Figs F12–F14).

We also investigated the benefit of our cell‐specific approach by building a model using averaged experimental data from all nine cells instead. This approach is described in Supporting information, Appendix F and the results are summarised in Supporting information, Appendix Table F12. Generally, for the cells with the highest data quality (Cells 1–5) the cell‐specific models provided better predictions than the average model, as can be seen for Pr3 when comparing coloured cell‐specific predictions and experiment with the black line for the average model in Fig. 7 *B*. The same trend held for the action potential protocol Pr6: in 8/9 cells the cell‐specific model provided less error than the average cell model – the largest improvement was 50% less error; for the remaining cell where the average cell model provided better predictions, this was by 3%.

## Discussion

In this paper we have presented a novel method for capturing ion current properties, based on constructing mathematical models of ion channel kinetics. We used a sinusoidal voltage protocol to construct a simple model of hERG channel kinetics using just 8 s of recording, as opposed to a traditional approach that requires several minutes of voltage‐step data. All of our experimental data can be collected from a single cell, whereas traditional protocols require long experiments, and typically require different gating processes to be studied in different experiments in different cells. For the future, our approach opens up the possibility of making multiple interventions (such as the addition of drug compounds) since we could re‐measure the full ion channel kinetics multiple times in a single cell.

The conceptual shift is that channel kinetics should be summarised by mathematical model parameters, not a series of *I–V* and τ–*V* curves. In essence, the model is the current characterisation, rather than something designed to fit *I–V* and τ–*V* curves, which only represent a certain subset of possible behaviours of the current. The success of the approach lies in moving away from traditional protocols that can be easily interpreted by eye, which typically require the current to return to an equilibrium rest state between voltage steps. Instead, our protocol also probes non‐equilibrium ion channel behaviour by rapidly exploring time and voltage dependence and is interpreted through the fitting of a model for the whole current at once.

Our model is able to replicate the experimental training data very well (Fig. 4). This is often the point at which traditional approaches in the literature have stopped and concluded that a mathematical model is a good representation of ion channel kinetics (also true more generally for mathematical models of biological processes). Instead, we performed an extremely thorough evaluation of the model by testing its ability to predict the behaviour in response to a series of voltage clamp protocols it has not ‘seen before’ (both those traditionally used to characterise hERG channel kinetics and a new complicated series of action potential waveforms), all recorded from the same cell as the training data. We are not aware of such a thorough, physiologically relevant validation of an ion channel model having been performed before. Testing that we are able to predict the current response to a voltage pattern which may be observed in physiological or pathophysiological conditions is a particularly robust and useful way to validate a model, and critical if an *I*_Kr_ model is to be used to accurately predict cardiac electrical activity in both healthy and potentially arrhythmic situations.

The extremely good prediction from all our cell‐specific models of the response to the complex action potential protocol is particularly remarkable (Fig. 6). Cell‐to‐cell variability in ion channel kinetics was captured by fitting different underlying kinetic parameters. These parameter sets were shown to have modest variation, and this variation in kinetics was quantitatively predictive of variation observed in independent validation experiments (Fig. 7).

Cell‐specific predictions were particularly strong when using the highest quality data, highlighting the necessary data quality for constructing accurate and robust models of ion channel kinetics. The cell‐specific models outperformed a model constructed using averaged data from multiple cells/experiments, in line with the ‘failure of averaging’ discussed in Golowasch *et al*. (2002) and the problems of fitting to averaged summary curves outlined in Pathmanathan *et al*. (2015). Our inactivation protocol (Pr4) showed that it is possible for models to fit some (or all) summary curves well, without necessarily replicating the underlying current traces with less error. Often studies present just single summary curves in isolation, but we have seen how models can fit certain summary curves well, whilst fitting others badly. Models that have less accurate summary curves may even predict the underlying current traces more reliably; and, importantly, vice‐versa.

A focus on these summary curves to represent kinetics and fit mathematical model behaviour was necessary in the era of hand fitting parameters using graph paper, but should perhaps now be superseded by fitting/comparing directly to experimental current traces. By fitting directly, we also reduce the possible influence of subjective choices during time constant fitting used in the generation of τ–*V* relationships.

A limitation of our study is that our model was trained on experiments performed in expression line cells, creating a hERG1a model at room temperature, compared to native *I*_Kr_ current in cardiac cells which will have additional isoforms, subunits and regulation at physiological temperatures. As a result, we do not state that this ion current model would necessarily give better performance within a cardiac action potential model. To characterise native *I*_Kr_ kinetics we plan to apply the methodology presented here in myocytes, to make a model that is more applicable for use in cardiac safety testing and whole‐organ simulations. The presence of many larger voltage‐dependent currents than we observe in expression systems will make this challenging, but a dofetilide subtraction approach may still yield good results.

There are still some aspects of the experimental behaviour that are not replicated by our model. These aspects may be a consequence of using a simple Hodgkin–Huxley‐style model formulation, although it remains a commonly used structure for currents within action potential models. In particular, there is only one time constant of deactivation, and low voltage‐dependence in the inactivation time constant (Fig. 5). A more complicated model with additional states and parameters may be needed to capture certain behaviours.

We assessed the capability of the protocol to fit a more complex five‐state Markov model for hERG (the model proposed by Wang *et al*. 1997), and show the results in Supporting information, Appendix H. Previously, Bett *et al*. (2011) explored the behaviours of a subset of existing hERG models and concluded that this model was best able to replicate activation kinetics. In Supporting information, Appendix H we show that exactly the same approach and algorithms again tightly constrained all 15 of the parameters in this larger model, using the same sinusoidal protocol data. The more complex model resulted in a better fit to the calibration data, and also made good predictions for the validation protocols – although not quite as good as the simpler model presented here in the main text. This finding highlights the importance and challenges of selecting the most appropriate level of complexity for a mathematical model.

So despite our simple model not replicating precisely the full range of behaviour, neither do the existing, more complex, models available in the literature. We have shown that our simple model can provide better predictions than the literature models for all the raw current time courses, if not all summary curves, in the majority of cells. In fact, the simplicity of our model may be the key to its success – with only eight kinetic parameters we have confidence that they are all being fitted well, and we have shown that there is low uncertainty in their values.

The applicability of our approach for different ion channels will be heavily dependent on the precise form of the sinusoidal protocol that is used, and in parallel work we are developing different strategies for optimising the voltage protocol design for given currents. Although we have also shown that the existing protocol is at least theoretically appropriate for parameterising an *I*_Ks_ model in Supporting information, Appendix G. In future work, ideas from control engineering may be useful. Seemingly unconnected problems, such as generating signals to characterise the state of lithium ion batteries (Xiong *et al*. 2011), are in fact very similar mathematical challenges.

There will be limits in the complexity of model structure and number of parameters that any protocol can constrain. But in terms of limitations of this style of protocol, we consider that the more information‐rich protocols are, the better; and these new protocols may enable us to accurately calibrate larger models than before. We strongly advocate synthetic data studies to assess the suitability of a given protocol for constraining parameters of a given model – seeing whether re‐fitting to data generated by simulations of a model and protocol can recover the parameters used in the simulation. Such approaches are necessary but not sufficient: they still rely on the models being a good representation of the system under study, and incorporating statistical ideas to handle model discrepancy (the difference between models and reality) is an important line of enquiry (Strong *et al*. 2012). In other parallel work, we are extending the approach presented here for selecting between different possible model structures for hERG channel kinetics (see Supporting information, Appendix Fig. A1 for the range of possibilities and Kargol (2013) for an outline of how this may be approached by optimising the protocols themselves to assist with this task).

Considering probabilistic uncertainty in model parameters and predictions is ever more important as models begin to be used for safety‐critical predictions (Pathmanathan & Gray, 2013; Mirams *et al*. 2016). These predictions include guiding therapies (Arevalo *et al*. 2016) and pharmaceutical safety assessment with the Comprehensive in‐vitro Proarrhythmia Assay initiative being pursued by the Food and Drug Administration in collaboration with industry, academia and other regulators (Sager *et al*. 2014; Fermini *et al*. 2016). Here we have shown there is very low uncertainty in hERG kinetics parameters in a single cell, and also characterised the variability in these estimates between different cells.

In summary, we have demonstrated significant advantages in our cell‐specific mathematical modelling approach, observing excellent model predictions of currents in response to protocols the model was not trained to replicate. The simple ion channel state arrangement we have assumed must capture the most important features underlying hERG state transitions, despite being much simpler than many previous models in the literature. The information‐rich approach allows, for perhaps the first time, an exploration of both within‐cell and between‐cell variability in ion channel kinetics. The significant time saving of our short protocol also leads to datasets that are more consistent and therefore of higher quality, since little changes in experimental conditions during the 8 s recording interval. Its brevity opens up the possibility of taking more recordings in different experimental conditions within a single cell (e.g. drug concentrations (Lee *et al*. 2016; Pearlstein *et al*. 2016) or temperatures (Vandenberg *et al*. 2006)). These datasets will result in more accurate descriptions of ionic currents in these different conditions in the heart and other organ systems.

## Additional information

### Competing interests

The authors declare that the research was conducted in the absence of any commercial or financial relationships that could be construed as a potential conflict of interest. The opinions presented here are those of the authors. No official support or endorsement by the Food and Drug Administration is intended nor should be inferred.

### Author contributions

K.A.B., R.B., Y.C., D.J.G., T.deB. and G.R.M. designed the study and modelling approach; K.A.B., R.B. and G.R.M. designed and implemented the statistical methods; K.A.B., G.R.M., J.I.V., A.P.H. and T.deB. designed and refined the experimental methods; K.A.B. performed all the experiments, simulations and statistical analysis; K.A.B., T.deB. and G.R.M. wrote the manuscript. All authors have read and approved the final version of this manuscript and agree to be accountable for all aspects of the work in ensuring that questions related to the accuracy or integrity of any part of the work are appropriately investigated and resolved. All persons designated as authors qualify for authorship, and all those who qualify for authorship are listed.

### Funding

This work was supported by the UK Engineering and Physical Sciences Research Council [grant numbers EP/G037280/1, EP/I017909/1, EP/K503769/1]; the Wellcome Trust [grant number 101222/Z/13/Z] and the Royal Society through a Sir Henry Dale Fellowship to GRM; the Australia NHMRC; and the France ANR [grant number BoB ANR‐16‐CE23‐0003].

### Data deposition

All computational codes, and the experimental current recordings that were used for calibration and validation (leak and dofetilide subtracted), are openly available in a Supplementary data repository at https://github.com/mirams/sine-wave. A permanently archived version is available on Figshare at https://doi.org/10.6084/m9.figshare.4704550.v5 alongside the full raw data (in both plain text and pCLAMP formats) at https://doi.org/10.6084/m9.figshare.4702546.v1. For additional details on the methods, see Supporting information, Appendix.

## Supporting information

Disclaimer: Supporting information has been peer‐reviewed but not copyedited.

**Appendix A**. Details of published hERG channel models.
**Appendix B**. Additional methods.
**Appendix C**. Synthetic data study to assess protocol information content.
**Appendix D**. Cell‐specific *versus* literature model predictions.
**Appendix E**. Additional current–voltage relationship predictions.
**Appendix F**. Comparing cell‐specific with average model.
**Appendix G**. Synthetic data study for an *I*_Ks_ model.
**Appendix H**. Testing with a five‐state Markov model.
