# Supplementary material for: Sinusoidal voltage protocols for rapid characterisation of ion channel kinetics
Source: J Physiol. 2018 Apr 17;596(10):1813–28. doi: 10.1113/JP275733 (PMC5978315; doi:10.1113/JP275733)
Supplement: Supplementary file 1 — Appendix A. Details of published hERG channel models. Appendix B. Additional methods. Appendix C. Synthetic data study to assess protocol information content. Appendix D. Cell‐specific versus literature model predictions. Appendix E. Additional current–voltage relationship predictions. Appendix F. Comparing cell‐specific with average model. Appendix G. Synthetic data study for an I Ks model. Appendix H. Testing with a five‐state Markov model. [file TJP-596-1813-s001.pdf]

Sinusoidal voltage protocols for rapid characterisation of ion channel kinetics: Appendix

Kylie A. Beattie, Adam P. Hill, Rémi Bardenet, Yi Cui, Jamie I. Vandenberg, David J. Gavaghan, Teun P. de Boer, Gary R. Mirams

January 31, 2018

Contents

A Details of Published hERG Channel Models **2**

B Additional Methods **4**
- B1 Additional Experimental Methods . . . 4
  - B1.1 Cell Culture . . . 4
  - B1.2 Electrophysiology Solutions . . . 4
  - B1.3 Recording Techniques . . . 4
  - B1.4 Details of Voltage Clamp Protocols . . . 4
  - B1.5 Liquid Junction Potential . . . 6
  - B1.6 Effect of Dofetilide Subtraction . . . 6
  - B1.7 Deriving IV Curves and Time Constant-V Curves . . . 7
- B2 Bayesian Inference Scheme . . . 8
  - B2.1 Likelihood formulation . . . 8
  - B2.2 Conductance estimation to inform the prior . . . 10
  - B2.3 Prior . . . 10
  - B2.4 Global minimisation . . . 11
  - B2.5 Markov Chain Monte Carlo parameter inference . . . 11

C Synthetic Data Study to Assess Protocol Information Content **13**
- C1 Producing synthetic data . . . 13
- C2 Inferring parameters from synthetic data . . . 13

D Cell-Specific versus Literature Model Predictions **14**

E Additional Current-Voltage Relationship Predictions **18**

F Comparing Cell-Specific with Average Model **20**

G Synthetic data study for an I_{Ks} model **26**

H Testing with a five state Markov model **27**

A Details of Published hERG Channel Models

Figure 1 of the main text features simulations from 29 literature hERG or I_{Kr} models. In Table A1 we list these models, give references, and show the seven different structures that they feature in Figure A1.

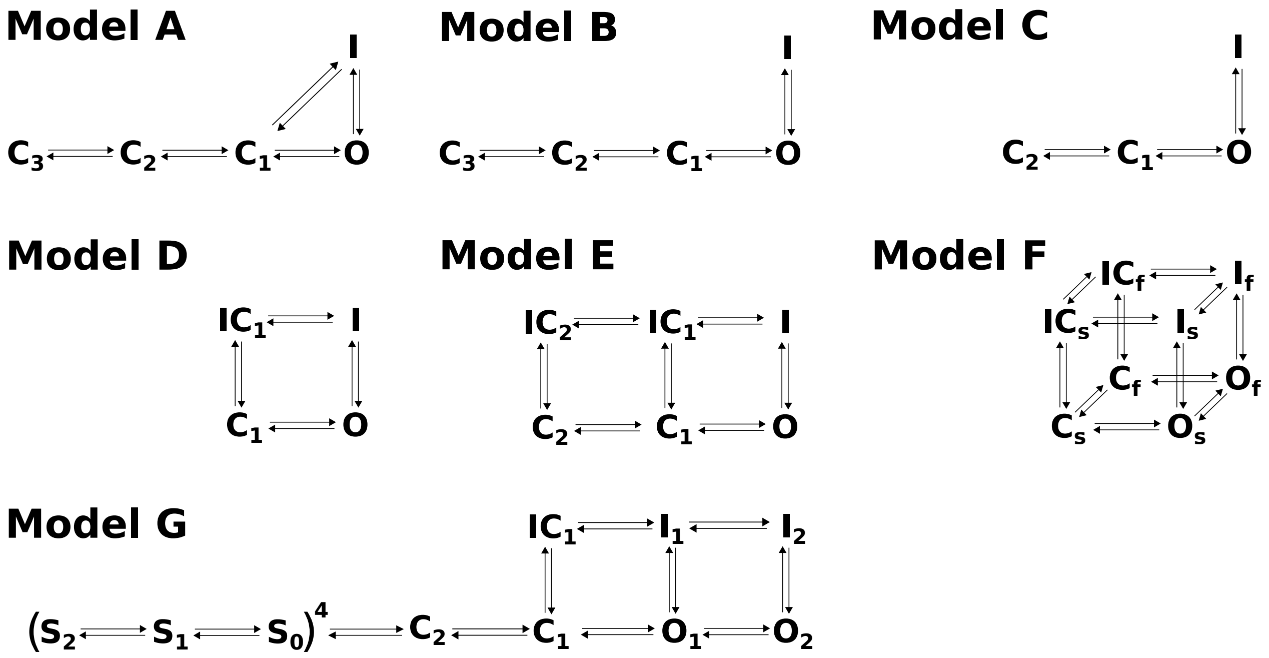

Figure A1: Different mathematical model structures for the literature models listed in Table A1. The model we use in the main text takes structure D as shown in Figure 4B. Note that, depending on their parameterisations, models D, E, F and G could satisfy independent gating (Hodgkin-Huxley assumptions) and then the states annotated as 'I' could also be considered 'IO' — but as drawn here this is not a requirement, and in neither case does current flow, so we removed the 'O' from these for simplicity.

There are two models in this table that are not in Figure 1: the Kiehn et al. (1999) model as it is defined only at certain voltages; and the Piper et al. (2003) model as it does not easily fit into the Hodgkin-Huxley/Markov model framework we used in our simulation code.

Table A1: Table summarising details of each published I_{Kr} model formulation, in chronological order. '*# Params*' indicates the total number of free parameters (the number given includes a G_{Kr} parameter for the conductance). The *Model Type* is 'HH' for Hodgkin–Huxley models and 'MM' for Markov Models, or a hybrid of the two (MM/HH, which generally means a MM with some symmetry in transition rates). *Temp.* represents temperature and is given as 'Room' for room temperature or 'PT' for physiological temperature. *Structure* respresents the model structure as shown iin Figure A1 We see that models are calibrated to experimental datasets from different conditions — species, cell types and temperatures. These conditions may not be the same temperature, cell type or even species as the models are intended to represent (see Niederer et al., 2009), and so the final column indicates the species of the cell model in which the hERG model was used.

Model	Model Type	# Params	Experimental Calibration Data Conditions: Species and Cell Type(s)	Temp.	Structure	Model for Species
Zeng et al. (1995)	HH	11	Guinea pig ventricular myocytes	PT	D	Guinea pig
Lindblad et al. (1996)	HH	11	Rabbit SA, AV, atrial myocytes and rabbit and guinea pig ventricular myocytes	PT	D	Rabbit
Liu et al. (1996)	MM	11	Ferret atrial myocytes	Room	C	Ferret
Wang et al. (1997)	MM	15	*Xenopus* oocytes	Room	B	N/A
Courtemanche et al. (1998)	HH	10	Human atrial myocytes	PT	D	Human
Nygren et al. (1998)	HH	9	Human atrial/rabbit atrial myocytes and *Xenopus* oocytes	PT	D	Human
Priebe and Beuckelmann (1998)	HH	9	Human ventricular myocytes	PT	D	Human
Kiehn et al. (1999)	MM	9*	*Xenopus* oocytes	Room	A	N/A
Winslow et al. (1999)	HH	7	Guinea pig ventricular myocytes	PT	D	Dog
Ramirez et al. (2000)	HH	13	Canine atrial myocytes	PT	D	Dog
Zhang et al. (2000)	HH	15	Rabbit sino-atrial node	PT	F	Rabbit
Clancy and Rudy (2001)	MM	14	Guinea pig ventricular	PT	A	Guinea pig
Lu et al. (2001)	MM	17	Chinese Hamster Ovary (CHO)	PT	A	N/A
Mazhari et al. (2001)	MM	17	Human Embryonic Kidney (HEK) 293	PT	A	N/A
Fox et al. (2002)	HH	10	Canine ventricular myocytes	PT	D	Dog
Kurata et al. (2002)	HH	18	Rabbit sino-atrial node	PT	F	Rabbit
Oehmen et al. (2002)	MM	11	Rabbit sino-atrial node	PT	C	Rabbit
Matsuoka et al. (2003)	HH	23	Rabbit sino-atrial node and guinea pig ventricular myocytes	PT	F	Guinea pig
Piper et al. (2003)	MM/HH	43	*Xenopus* oocytes	Room	G	N/A
Seemann et al. (2003)	HH	7	Human ventricular myocytes	PT	D	Human
Hund and Rudy (2004)	HH	11	Canine ventricular myocytes	PT	D	Dog
Shannon et al. (2004)	HH	11	Rabbit ventricular myocytes	PT	D	Rabbit
Ten Tusscher et al. (2004)	HH	13	HEK 293/CHO/*Xenopus* oocytes	PT	D	Human
Fink et al. (2008)	MM	15	Human Embryonic Kidney (HEK) 293	PT	B	Human
Aslanidi et al. (2009)	HH	8	Canine Purkinje	PT	D	Dog
Inada et al. (2009)	HH	20	Rabbit atrio-ventricular node	PT	F	Rabbit
Grandi et al. (2010)	HH	12	Human ventricular myocytes	PT	D	Human
O'Hara et al. (2011)	HH	19	Human ventricular myocytes	PT	F	Human
Severi et al. (2012)	HH	17	Rabbit sino-atrial node	PT	F	Rabbit
Di Veroli et al. (2013)	MM/HH	13	Chinese Hamster Ovary (CHO)	Room	E	N/A
Di Veroli et al. (2013)	HH	17	Human Embryonic Kidney (HEK) 293 expressing canine ERG	PT	D	N/A

* The transition rates of the Kiehn et al. (1999) model are defined at specific voltages, so for this model there are 8 parameters (and 1 conductance parameter) for each voltage at which the model is defined.

B Additional Methods

This section contains further description of the methods that were used, with a particular focus on details of the Bayesian Inference scheme in Section B2. These sections do not feature in the main Methods section due to space constraints.

B1 Additional Experimental Methods

B1.1 Cell Culture

Chinese Hamster Ovary (CHO) cells stably expressing Kv11.1 were used in the patch clamp experiments performed in this study. Cells were cultured in Ham's F12 nutrient mix containing 5% foetal bovine serum and maintained at 37°C with 5% CO_2.

B1.2 Electrophysiology Solutions

The bath solution was composed of: NaCl (137 mM), KCl (4 mM), $MgCl_2$ (1 mM), HEPES (10 mM), glucose (10 mM), and $CaCl_2$ (1.8 mM). The pH of the solution was adjusted to 7.4 with NaOH. Borosilicate glass micropipettes were pulled and fire polished to final tip resistances of approximately 2–5 MΩ when filled with pipette solution containing: KCl (130 mM), $MgCl_2$ (1 mM), HEPES (10 mM), EGTA (5 mM), and MgATP (5 mM). pH of the solution was adjusted to 7.2 with KOH. All experiments were performed at room temperature (21–22°C). Using this temperature and the composition of the bath and pipette solutions, a K^+ reversal potential of approximately -88.4 mV was calculated using the Nernst potential (equation (8)), the exact value depending on the particular temperature of each experimental recording.

B1.3 Recording Techniques

Current recordings were made using an Axopatch 200B amplifier in whole-cell patch clamp mode. Data acquisition was performed using pClamp 10 software (Molecular Devices, Sunnyvale, USA). The protocols were first created as text files and then converted to `.abf` stimulus files to make corresponding `.pro` protocol files in the pClamp 10 software. A CV 203BU amplifier headstage and a Digidata 1440A were used. A Sutter MP225 micromanipulator was used for positioning of the microelectrode. The current signal was sampled at a rate of 10 kHz. 75–80% series resistance compensation was applied and data were 5 kHz low pass Bessel filtered by the hardware. No software filtering was applied. Whole-cell capacitance compensation was applied electronically. Leak subtraction was applied offline by using a 50 ms leak step to allow correction. To make a series of successive recordings using different protocols on the same cell, the pClamp "Sequencing Keys" tool was utilised, with a `.sks` file detailing the sequence the protocols should be performed in.

B1.4 Details of Voltage Clamp Protocols

Here we list the details of the voltage-clamp protocols that were not included in the main text. The protocols can also be found encoded in the software, available to download as described at the end of the main text.

Protocol 0 — Repeated activation step

Before the start of each set of recordings on each cell an activation step protocol with a start-to-start interval of 12 seconds was repeated several times until consistent currents were observed on

each repeat. From an initial holding potential of −80 mV, this protocol comprised a 5 s step to 10 mV followed by a 5 s step to −50 mV before returning again to a holding potential of −80 mV. This protocol is depicted in Figure B2. We repeated this protocol while dofetilide was added (see Figure 2) and the current traces recorded from this protocol were used to assess when a steady level of dofetilide block had been reached.

Protocols 1,2 — Activation Kinetics

After the initial period at holding potential incorporating the −120 mV leak step, a step to V_{step_1} followed and was held at that voltage for T_{step} ms, before a step to −120 mV for 2.5 s, before returning to holding potential of −80 mV for 1 second. The protocol was repeated 6 times with a different T_{step} on each repeat. T_{step} took the values of 3, 10, 30, 100, 300 and 1000 ms.

- For Protocol 1, V_{step_1} is 0 mV. This protocol is depicted in Figure B3.
- For Protocol 2, V_{step_1} is +40 mV. This protocol is depicted in Figure B4.

Figures B2–B4 show plots of the voltage clamps that are used in the repeated activation step and activation kinetics protocols (Pr0–Pr2).

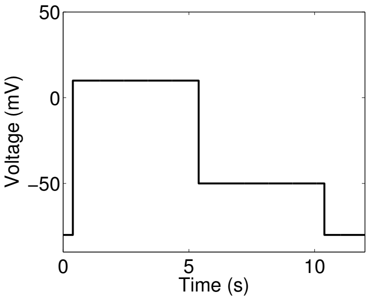

Figure B2: Repeated Activation Step Protocol (Pr0).

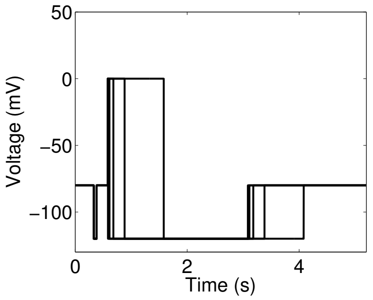

Figure B3: Activation Kinetics 1 Protocol (Pr1).

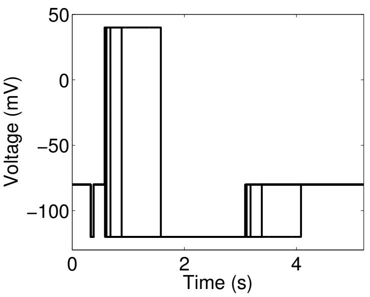

Figure B4: Activation Kinetics 2 Protocol (Pr2).

Protocol 3 — Steady-State Activation

From the initial period at holding potential incorporating the −120 mV leak step, a step to V_{step} was applied for 5 seconds, followed by a 1 s step to −40 mV, before a 500 ms step to −120 mV, and then returning back to holding potential for one second. This process was repeated 7 times with a different V_{step} on each repeat. V_{step} ranged from −60 mV to +60 mV in 20 mV increments. This protocol is depicted in Figure 5A (left column).

Protocol 4 — Inactivation

From the initial period at holding potential incorporating the −120 mV leak step, a step to 50 mV for 600 ms, and a step to −90 mV for 60 ms, followed by a step to V_{step} for 150 ms, before a 500 ms step to −120 mV, and a 1 s step back to holding potential of −80 mV; This was repeated 16 times with a different V_{step} on each repeat. V_{step} ranged from −100 mV to 50 mV in 10 mV increments. This protocol is depicted in Figure 5A (middle column).

Protocol 5 — Deactivation

From the initial period at holding potential incorporating the −120 mV leak step, a step to 50 mV for 2 s was applied, followed by a step to V_{step} for 6 s, before a 500 ms step to −120 mV, and then returning back to holding potential for one second. This process was repeated 9 times with a different V_{step} on each repeat. V_{step} ranged from −120 mV to −40 mV in 10 mV increments. This protocol is depicted in Figure 5A (right column).

Protocol 6 — Action Potentials Clamp

See main text.

Protocol 7 — Sinusoidal Clamp

The full protocol is comprised of 250 ms at holding potential of −80 mV, followed by a 50 ms 'leak detection' step to −120 mV, and then 200 ms back at −80 mV. This was followed by an 'activation' step of 1 s step to 40 mV; a 'closing' 500 ms step to −120 mV; and a return to −80 mV for 1 second. The 3.5 s sinusoidal portion of the protocol then followed (the form of which is described below), before a 'closing' 500 ms step to −120 mV, and a return to −80 mV for 1 s.

The sinusoidal portion of the protocol takes the form of a sum of three sine waves as discussed in the main text Equation (1), and repeated here for convenience:

$$V(t) = -30 + A_1 \sin\left(\omega_1(t - t_0)\right) + A_2 \sin(\omega_2(t - t_0)) + A_3 \sin(\omega_3(t - t_0)),$$

where $A_1 = 54\,\text{mV}$, $A_2 = 26\,\text{mV}$, $A_3 = 10\,\text{mV}$, $\omega_1 = 0.007\,\text{ms}^{-1}$, $\omega_2 = 0.037\,\text{ms}^{-1}$ and $\omega_3 = 0.19\,\text{ms}^{-1}$, and t is time measured in milliseconds.

The protocol was initially designed with just the −120 mV leak step and not the additional 'activation' steps to 40 mV and −120 mV (which were included after preliminary experiments as described in Section B2.2) and so the sine wave was shifted using $t_0 = 2500\,\text{ms}$ to begin at the same phase after we incorporated the additional steps. This offset is not expected to be important to include, but was included here for clarity for anyone attempting to reproduce our study.

B1.5 Liquid Junction Potential

All of the protocols described in this section were adjusted on the amplifier to account for the liquid junction potential which was calculated to be 4.1 mV from the ionic composition of our physiological solutions which are described in Section B1.2. The liquid junction potential was calculated using the junction potential calculator in the pClamp software.

B1.6 Effect of Dofetilide Subtraction

To show the effect of 0.3 μM dofetilide subtraction and to demonstrate the lack of endogenous currents remaining following this step, we compare the current traces before and after dofetilide subtraction in Figure B5. We show currents from Cell #5 which features in the figures of the main text.

There was little contribution from endogenous currents in these cells. The application of 0.3 μM dofetilide eliminates almost all current, indicating that both the vehicle recording and 'dofetilide subtracted' currents are almost entirely due to hERG. While levels of endogenous currents and the impact of leak subtraction on the current traces may vary from cell-to-cell, overall we found only small endogenous currents were observed in the cells.

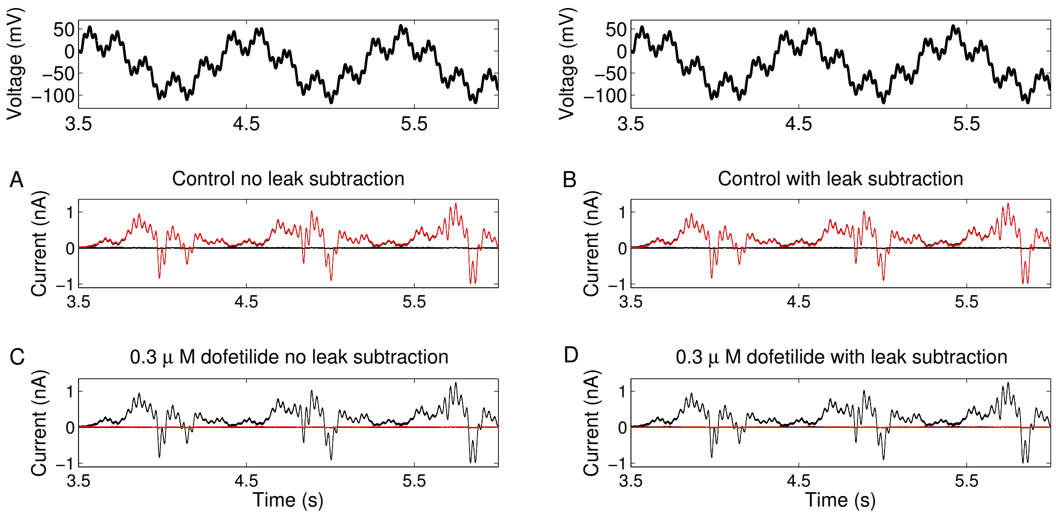

Figure B5: Raw recordings, recordings with leak subtraction, and 0.3 μM dofetilide controls for Cell #5. Top row: input voltage trace from the sine wave protocol (same on both sides for comparison with traces below). In A–D we show the current in response to this voltage protocol in four situations — each panel shows the same four traces, with a different one highlighted in red. In A we highlight the raw recording of whole current; in B the whole current recording after leak subtraction, in C the raw recording after the addition of dofetilide; and in D the dofetilide recording after leak subtraction.

An example of where dofetilide subtraction may play a more important role is shown in Figure B6, where the hERG current is much lower and so any contribution from endogenous currents would have more impact and it may be important to remove this contribution. This example is for Cell #6 from the main text.

B1.7 Deriving IV Curves and Time Constant-V Curves

To derive time constant-voltage relationships from experimental data and simulated data traces, we used the Levenberg-Marquardt algorithm with a tolerance of 10^{-6} within Clampfit v10.5. To derive the instantaneous inactivation time constant curves shown in Figure 5 (inactivation column, row D) we fitted a single a single exponential to the current responses during the 150 ms V_{step}, as defined in the inactivation protocol (Pr4) description above.

To produce the deactivation and recovery from inactivation rate time constant–voltage relationship for the experimental data traces, we fitted a triple exponential through the experimental data trace from the deactivation protocol (Pr 5). The section of the data used for fitting is the current in response to the 6 second V_{step}. Both this region of experimental data used for fitting and that for the instantaneous inactivation time constant described above are highlighted in row B of Figure 5. The fastest time constant from the triple exponential fit to each test step corresponded to the recovery from inactivation time constant. We then used the weights of the remaining two time constants from each triple exponential fit to produce a single weighted time constant for deactivation (Lacroix et al., 2011). To derive the deactivation and recovery from inactivation time constants from simulated data we fitted a double exponential through the current in response to the 6 second

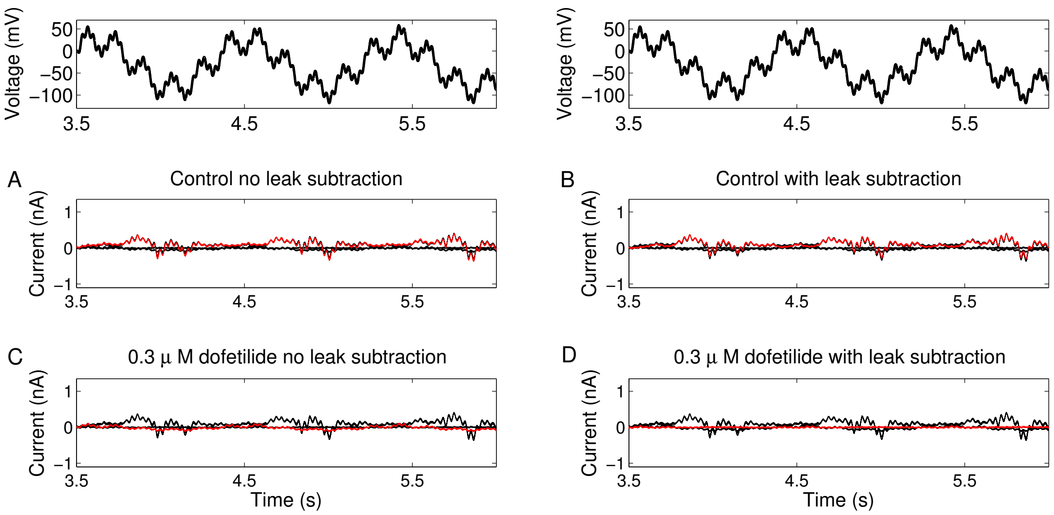

Figure B6: Raw recordings, recordings with leak subtraction, and 0.3 μM dofetilide controls for Cell #6. Top row: input voltage trace from the sine wave protocol (same on both sides for comparison with traces below). In A–D we show the current in response to this voltage protocol in four situations — each panel shows the same four traces, with a different one highlighted in red. In A we highlight the raw recording of whole current; in B the whole current recording after leak subtraction, in C the raw recording after the addition of dofetilide; and in D the dofetilide recording after leak subtraction.

V_{step} section of the deactivation protocol. Again, we used the faster time constant as the recovery from inactivation time constant and the slower time constant as that for deactivation.

To produce the peak current-voltage relationship for the steady state activation protocol for the simulated data traces we wrote MatLab code to identify the peak current in the region between 5.6292 and 5.7292 seconds on each sweep of the protocol, which corresponds to the current response just after the 5 second V_{step} when the voltage is stepped to -40 mV. We then normalised the peak current data to the maximum overall peak identified in this region to produce the current-voltage relationship curve. For the simulated data we wrote a MatLab script (included in code download) to identify the peak-current voltage relationship for this protocol but for the experimental traces we verified these peak points manually to avoid incorrect peaks being identified due to noise or capacitive effects. We also identified the peak currents in the currents evoked by the activation kinetics protocols manually for the same reason. In the activation kinetics protocol we identified the peak currents during the V_{step} for each interval of T_{step} duration.

B2 Bayesian Inference Scheme

B2.1 Likelihood formulation

For an observed experimental recording which we will denote $\mathbf{y}$, we can infer the probability of different combinations of model parameters θ. Bayes' rule underpins this approach which is expressed as

$$P(\theta|\mathbf{y}) = \frac{P(\mathbf{y}|\theta)P(\theta)}{P(\mathbf{y})} \tag{B.1}$$

$P(\theta|\mathbf{y})$ is a probability density that encodes our belief that the parameters of the model are in a neighbourhood of θ after observing the experimental data $\mathbf{y}$, and is termed the *posterior probability density*. $P(\mathbf{y}|\theta)$ is the probability density that corresponds to the probabilistic generation of the experimental data $\mathbf{y}$ given a model parameterised with parameters θ. $P(\theta)$ encapsulates our beliefs about θ before observing any experimental data and is termed the *prior distribution* (details of the prior that we used are in Appendix B2.3). $P(\mathbf{y})$ is a normalising term which is the integral of all possible probabilities $P(\mathbf{y}|\theta)$ and ensures that the posterior density $P(\theta|\mathbf{y})$ integrates to 1. In practice this normalising term is calculated by

$$P(\mathbf{y}) = \int P(\mathbf{y}|\theta)P(\theta)\mathrm{d}\theta. \tag{B.2}$$

A Bayesian inference approach to parameter estimation combines beliefs about the parameters in the prior distribution $P(\theta)$ with the *likelihood* $P(\mathbf{y}|\theta)$ to determine the posterior probability distribution $P(\theta|\mathbf{y})$.

We define the likelihood

$$L(\theta|\mathbf{y}) = P(\mathbf{y}|\theta) \tag{B.3}$$

to insist on the fact that we consider it as a function of θ, with $\mathbf{y}$ kept fixed at the observation values. Bayes' rule (in Equation (B.1)) can be rewritten in terms of likelihood as

$$P(\theta|\mathbf{y}) \propto P(\theta)L(\theta|\mathbf{y}). \tag{B.4}$$

When the prior distribution is assumed to be uniform (as it is in this study), we can make inferences based on just the likelihood, as the prior $P(\theta)$ is either constant or zero. If a proposed parameter is outside our chosen prior then likelihood is 0 and we simply record that this parameter set has a likelihood of 0 and propose another parameter set.

We assume that the errors at each time point are independent and so the conditional probability density of observing the whole experimental trace from time sample 0 to time sample T given the model parameter set θ is

$$L(\theta|\mathbf{y}) = \prod_{t=0}^{T} P(y_t|\theta). \tag{B.5}$$

We assume that the experimental noise is independently and normally distributed with a mean of zero and variance of σ^2. The likelihood is then expressed as

$$L(\theta|\mathbf{y}) = \prod_{t=0}^{T} \mathcal{N}(y_t|f_t(\theta), \sigma^2) = \prod_{t=0}^{T} \frac{1}{\sqrt{2\pi\sigma^2}} \exp\left(-\frac{(y_t - f_t(\theta))^2}{2\sigma^2}\right). \tag{B.6}$$

In our case $f_t(\theta)$ is the predicted current at each time point given the parameters, this is given by equation (7) after solving the model system (equations (3)–(6)). Calculating equation (B.6) requires the evaluation of the product of many numbers less than 1, so it is more numerically convenient to calculate the *log-likelihood* instead. As our aim is to identify parameter sets θ which maximise the likelihood in equation (B.6), maximising the likelihood is equivalent to maximising the log-likelihood:

$$\log\left(L(\theta|\mathbf{y})\right) = -\frac{1}{2}\sum_{t=0}^{T} \log(2\pi\sigma^2) - \frac{1}{2}\sum_{t=0}^{T} \frac{(y_t - f_t(\theta))^2}{\sigma^2}. \tag{B.7}$$

In practice, the sums over time in equation (B.7) are formulated so that we exclude time points from regions where the data are affected by capacitive spikes. To be precise, we exclude 5 ms

intervals following step-changes in the imposed voltage clamp. In the sine wave protocol (Pr7) these step-changes occur at 0.25 seconds, 0.3 seconds, 0.5 seconds, 1.5 seconds, 2 seconds, 3 seconds, 6.5 seconds and 7 seconds (spikes are seen in experimental recordings at these times in Figure 3).

B2.2 Conductance estimation to inform the prior

Preliminary work revealed that using sine wave protocols alone often allowed kinetic parameters in the hERG model to be recovered, but there was potential for identifiability problems (or at least we encountered difficulties in finding a global optimum due to a rugged likelihood surface) when simultaneously fitting the conductance parameter and transition rate parameters P_1 to P_8 (although previous work suggests all parameters are theoretically identifiable (Walch and Eisenberg, 2016)). To add extra information on conductance, we incorporated a voltage-step to $+40\,\text{mV}$ followed by a step down to $-120\,\text{mV}$, as described in the definition of the sinusoidal protocol above. The aim being to provoke a large current. We then fitted a single exponential through the slow time constant of the tail current exhibited during the $-120\,\text{mV}$ step (fitting was performed in the Clampfit software, using the Levenberg-Marquardt algorithm with a tolerance of 10^{-6}). We then extrapolated back to the point at which the voltage step to $-120\,\text{mV}$ was made, and used the extrapolated current value at this point to estimate a conductance at this time point (this extrapolation method is described in Vandenberg et al. (2012)). The conductance we estimated was used as a lower bound for the prior distribution of the conductance, as we describe below.

B2.3 Prior

In this section we describe our prior assumptions on the values that each model parameter can take. The prior for the conductance G_{Kr} is assumed to be independent of the kinetic parameters, and to take a uniform distribution. As discussed above, the lower bound is formed by estimating a lower bound on the conductance value 'directly' from the experimental data; the upper bound is assumed to be 10 times the value of the lower bound.

The other model parameters are within transition rates of the form

$$k = A \exp(BV), \tag{B.8}$$

where V is voltage and A and B are model parameters (P_1 to P_8 for k_1 to k_4, as shown in Figure 4).

For parameters of the form A we assumed that the prior distribution is uniform between 10^{-7} and 1000 ms^{-1}, again to cover (and extend beyond) the full physiological range expected with hERG channel gating.

We assume that the prior distributions for B parameters are uniform between 10^{-7} and 0.4 mV^{-1}. The lower bound for this parameter was selected as the voltage-dependence becomes practically redundant when B becomes small: when $B = 10^{-7}$ the value of $\exp(BV)$ will change by less than 0.0015% across the voltages we reach in this study. The upper value is beyond the physiologically expected range.

We also impose a prior on the maximum rate of transition k between any states (maximum across the full voltage range in the protocol (that is from -120 to 58.25 mV)). If the maximum rate k is greater than $1000\,\text{ms}^{-1}$, or less than $1.67 \times 10^{-5}\,\text{ms}^{-1}$, the pair of parameter values that give rise to this are assigned prior probability zero (strictly, this is equivalent to defining 2D prior on A and B, but is easier to describe here, and code, as an additional constraint): the lower bound is based on the assumption that a transition is not physiologically realistic if it occurs over a time scale slower than one minute; the upper bound was decided based on the prior for the individual

parameters A and B in the transition rate expression and to prevent the transitions occurring over a time scale much faster than would be physiologically expected.

Note that our analysis is relatively insensitive to the precise form of the prior that is used as there are around 80,000 data points (8 s of 10 kHz samples) in the likelihood product calculation of Equation (B.5), which is then also in a product with the prior in Equation (B.4). So, effectively, each of the 10,000 data points has the same impact as the prior does on the posterior. Given our likelihood is extremely peaked around its maximum (Figure 4C), we have observed no notable influence of the shape of the prior, as long as the maximum posterior density point is well away from the limits described above — which it has been in all cases. Note that the same concept means that, in our case, the "maximum likelihood estimate" (MLE — parameter set that maximises Equation (B.5)) would be practically indistinguishable from the "maximum a posteriori estimator" (MAP — parameter set that maximises Equation (B.4)) even if we had a non-uniform prior.

B2.4 Global minimisation

The Covariance Matrix Adaptation — Evolution Strategy (CMA-ES) algorithm was used to perform an initial exploration of the surface of the posterior density, and to identify parameter sets which allow the model to fit the experimental data well. The tolerance used is 10^{-4} and all other settings are the defaults in MatLab implementation of CMA-ES v3.61, downloaded from `https://www.lri.fr/~hansen/cmaes.m`. We imposed bounds based on the prior as we describe above in Section B2.3.

We run the CMA-ES algorithm from different starting points and continue to do so until we identify the same region of parameter space for optimal parameter sets for each experimental data trace when starting from many different starting points. In this way, we can be confident that we identify the same region of high likelihood consistently (not simply the first local minimum that is found), and we have more confidence that this corresponds to the globally optimal likelihood.

These initial starting points for the CMA-ES algorithm are sampled from within the prior defined for each parameter, described in section B2.3. To sample from the prior we simply select the voltage-dependent transition rate parameters (of the form B described above) uniformly from the defined range. The same approach is used to sample the conductance parameter.

For the parameters of the form A above we sample starting points in a logarithmic fashion across the range of the uniform prior. This approach helps to restrict the initial guesses of parameters to the region of measurable time scales we imposed by defining the maximum and minimum ranges on the overall transition rate, as described above. We also run a small selection of starting points with both A and B parameter values sampled uniformly from $[10^{-7}, 0.1]$ (the range in which most existing model parameters lie), again to ensure we identify the global optimal solution to the optimisation problem. We log-transform all parameters within CMA-ES to aid the optimisation process by making all values similar orders of magnitude.

B2.5 Markov Chain Monte Carlo parameter inference

We use *Markov Chain Monte Carlo* (MCMC) methods to explore the posterior probability distribution. The approach we use is the *Metropolis-Hastings* algorithm. In this algorithm, candidate parameter sets are proposed from a *proposal distribution* $q(\theta_{\mathbf{cand}}|\theta_{\mathbf{i}})$ which depends only on the previously accepted parameter set $\theta_{\mathbf{i}}$. We use a multivariate normal distribution as our proposal distribution. Any candidate parameter set θ_{cand} is compared to the current parameter set θ_i by calculating the ratio of the likelihood of the two parameter sets. The value of the ratio determines whether or not the proposed parameter set is accepted as part of the MCMC chain. If the candidate parameter set has a greater posterior density value than the existing parameter set then

it will be added to the Markov chain, that is $\theta_{\mathbf{i+1}} = \theta_{\mathbf{cand}}$. Otherwise, the parameter set may still be accepted with a probability equal to the ratio of likelihood/posterior density values. That is, a proposed parameter set generated from a multivariate normal distribution is accepted with probability

$$\alpha = \min\left\{\frac{L(\theta_{\mathbf{cand}}|\mathbf{y})}{L(\theta_{\mathbf{i}}|\mathbf{y})}, 1\right\}. \tag{B.9}$$

Also note that if the proposed parameter set contains any parameters outside the range of the prior, or violates any of the conditions on the parameters that we have imposed, the parameter set is assigned an acceptance probability of 0 and immediately rejected and the previously accepted parameter set is again added to the Markov chain — that is, $\theta_{i+1} = \theta_i$.

In practice, we use a covariance matrix adaptive version of the Metropolis-Hastings Algorithm which helps identify the directions in parameter space which have the highest likelihood values, the algorithm is described in Haario et al. (2001). At each iteration of the algorithm, the covariance matrix of the multivariate normal distribution is updated and a scalar value is also updated to define the width of the distribution. We run our MCMC chains for 250,000 samples and discard the first 50,000 samples as 'burn in' (for an introduction to MCMC see Gilks et al. (1996)).

C Synthetic Data Study to Assess Protocol Information Content

In order to verify that there was sufficient information within the sinusoidal protocol to parameterise our model we performed a *synthetic data* study. The aim in such a study is to ascertain whether we can recover the parameters used in the simulation from a simulated data trace (with added noise in this case).

C1 Producing synthetic data

In order to produce synthetic data we simulate with some fixed known parameter set. We performed this with both our best initial parameter set estimate (those parameters in literature HH models), and also from the parameters we obtained after fitting to the experimental data trace as we present here (both showed good identifiability). We scale the simulated trace by multiplying by this factor, so it becomes approximately the same magnitude (in nA) as the experimental trace. We estimated the typical level of noise from the experimental trace by calculating the standard deviation σ of the experimental current during the first 200 ms (where the current is around zero at the initial holding potential of -80 mV). We then generate a synthetic data trace by adding normally distributed noise with a mean of zero and the standard deviation equal to the noise estimated from the experimental trace ($\sim N(0, \sigma^2)$) to the conductance-scaled simulated trace. The example we present here uses the experimental reference trace from Cell #5, featured in much of the manuscript.

C2 Inferring parameters from synthetic data

We then attempt to infer parameters from this synthetic data trace, using the CMA-ES algorithm followed by MCMC as described in Section 2.5. In Figure C7 we present probability density distributions obtained when using both synthetic and experimental traces. We are able to recover the original parameters underlying the synthetic trace with high accuracy.

The synthetic data study provides us with confidence in the suitability of our protocol for accurately identifying parameters of the model presented in Figure 4B in the main text, and also that the parameter inference protocol(s) we are using are suitable for the task. We believe such an approach should always be used to test whether there is sufficient information in the experimental data being proposed for calibration of a mathematical model. The test should be performed twice: before conducting the experiment (with the pre-existing best guess at the parameters); and also after conducting the experiment (with the new maximum posterior density estimate of the parameters — as we illustrate in Figure C7).

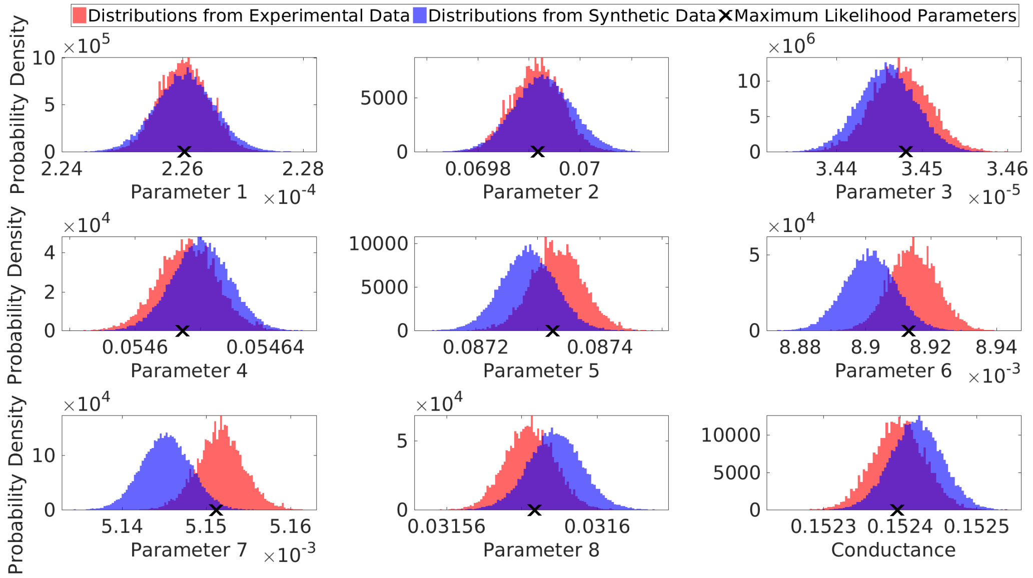

Figure C7: Probability density distributions for each parameter estimates from fitting to both experimental data (red) and simulated data (blue). Crosses indicate the parameter set with the maximum posterior density.

D Cell-Specific versus Literature Model Predictions

In Tables D2–D10 we compare the predictions given by each cell-specific model with a range of literature model predictions. We compare their ability to predict the full current traces for the validation protocols Pr3–6 discussed in the main text. Each table provides the mean (over each time point) square difference between an experimental current recording in one particular cell and its cell-specific model prediction under each of the validation protocols, and compares this with current predictions from a range of literature models. Equation (F.10) gives the formula that was used to calculate the error entries.

Note that we have to choose a conductance value, G_{Kr}, for the literature models. G_{Kr} is selected differently for each cell by minimising the error metric for the predicted current trace under the action potential protocol (Pr6) for each model (a best-case scenario for each literature model). Our new cell-specific models' conductances were fitted to the sine wave protocol (Pr7), along with the rest of their parameters. N.B. the literature model predictions are worse if we scale them to fit the sine wave; we considered this perhaps unjustified since they were developed never having seen such a protocol.

Despite literature models having their conductance scaled to minimise error in the Pr6 (action potential clamp) current prediction; only the Di Veroli et al. (2013) model for Cell #9 performs better than our cell-specific models. The sine-wave fitted model outperforms all other literature models for all other cells.

Additionally, the Wang et al. (1997) model gives better predictions for the deactivation protocol current for some cells and for the steady state activation protocol for Cell #4. The Di Veroli et al. (2013) model gives better predictions for the inactivation protocol for Cells #2, and #9; and the deactivation protocol for Cell #6. The Mazhari et al. (2001) model gives a better prediction for the steady state activation protocol for Cell #7.

Table D2: Table quantifying mean square difference (units nA) between experimental current traces and simulation predictions for the validation protocols shown in Figures 5 and 6 for Cell #1. Here the colour scale is set so that [yellow] represents zero error and [red] represents the highest error for each protocol/column.

Model	Sine Wave (Pr7)	AP (Pr6)	Steady Act. (Pr3)	Deact. (Pr5)	Inact. (Pr4)
New model for Cell #1	0.0151	0.0283	0.0332	0.0925	0.0312
Wang et al. (1997)	0.0389	0.0419	0.0842	0.1195	0.0402
Di Veroli et al. (2013)	0.0487	0.0545	0.0916	0.1400	0.0479
Mazhari et al. (2001)	0.0499	0.0516	0.0731	0.1491	0.0564
Ten Tusscher et al. (2004)	0.0599	0.0557	0.0939	0.1538	0.0653
Zeng et al. (1995)	0.0787	0.0802	0.0989	0.1616	0.0638

Table D3: Table quantifying mean square difference (units nA) between experimental current traces and simulation predictions for the validation protocols shown in Figures 5 and 6 for Cell #2. Here the colour scale is set so that [yellow] represents zero error and [red] represents the highest error for each protocol/column.

Model	Sine Wave (Pr7)	AP (Pr6)	Steady Act. (Pr3)	Deact. (Pr5)	Inact. (Pr4)
New model for Cell #2	0.0164	0.0295	0.0270	0.0656	0.0219
Wang et al. (1997)	0.0529	0.0528	0.0746	0.0633	0.0384
Di Veroli et al. (2013)	0.0314	0.0302	0.0506	0.0679	0.0212
Mazhari et al. (2001)	0.0361	0.0380	0.0533	0.0939	0.0324
Ten Tusscher et al. (2004)	0.0575	0.0639	0.0846	0.1011	0.0539
Zeng et al. (1995)	0.0654	0.0717	0.0828	0.1070	0.0452

Table D4: Table quantifying mean square difference (units nA) between experimental current traces and simulation predictions for the validation protocols shown in Figures 5 and 6 for Cell #3. Here the color scale is set so that [yellow] represents zero error and [red] represents the highest error for each protocol/column.

Model	Sine Wave (Pr7)	AP (Pr6)	Steady Act. (Pr3)	Deact. (Pr5)	Inact. (Pr4)
New model for Cell #3	0.0253	0.0415	0.0658	0.0785	0.0537
Wang et al. (1997)	0.0472	0.0492	0.0729	0.0648	0.0549
Di Veroli et al. (2013)	0.0630	0.0859	0.1136	0.1034	0.0743
Mazhari et al. (2001)	0.0627	0.0819	0.0910	0.1099	0.0835
Ten Tusscher et al. (2004)	0.0733	0.0712	0.0936	0.1101	0.0822
Zeng et al. (1995)	0.0972	0.1153	0.1216	0.1282	0.0868

Table D5: Table quantifying mean square difference (units nA) between experimental current traces and simulation predictions for the validation protocols shown in Figures 5 and 6 for Cell #4. Here the color scale is set so that ■ represents zero error and ■ represents the highest error for each protocol/column.

Model	Sine Wave (Pr7)	AP (Pr6)	Steady Act. (Pr3)	Deact. (Pr5)	Inact. (Pr4)
New model for Cell #4	0.0258	0.0359	0.0701	0.0878	0.0522
Wang et al. (1997)	0.0595	0.0443	0.0587	0.0506	0.0537
Di Veroli et al. (2013)	0.0429	0.0559	0.1045	0.0955	0.0596
Mazhari et al. (2001)	0.0472	0.0581	0.0709	0.1074	0.0681
Ten Tusscher et al. (2004)	0.0668	0.0741	0.0760	0.1030	0.0665
Zeng et al. (1995)	0.0832	0.1067	0.1079	0.1282	0.0733

Table D6: Table quantifying mean square difference (units nA) between experimental current traces and simulation predictions for the validation protocols shown in Figures 5 and 6 for Cell #5. Here the color scale is set so that ■ represents zero error and ■ represents the highest error for each protocol/column.

Model	Sine Wave (Pr7)	AP (Pr6)	Steady Act. (Pr3)	Deact. (Pr5)	Inact. (Pr4)
New model for Cell #5	0.0203	0.0453	0.0437	0.1317	0.0700
Wang et al. (1997)	0.0725	0.0764	0.1374	0.1611	0.0933
Di Veroli et al. (2013)	0.0675	0.0958	0.1148	0.1881	0.0735
Mazhari et al. (2001)	0.0824	0.0963	0.1009	0.2286	0.1012
Ten Tusscher et al. (2004)	0.1080	0.1260	0.1603	0.2422	0.1415
Zeng et al. (1995)	0.1318	0.1650	0.1620	0.2575	0.1259

Table D7: Table quantifying mean square difference (units nA) between experimental current traces and simulation predictions for the validation protocols shown in Figures 5 and 6 for Cell #6. Here the color scale is set so that ■ represents zero error and ■ represents the highest error for each protocol/column.

Model	Sine Wave (Pr7)	AP (Pr6)	Steady Act. (Pr3)	Deact. (Pr5)	Inact. (Pr4)
New model for Cell #6	0.0113	0.0216	0.0264	0.0504	0.0170
Wang et al. (1997)	0.0263	0.0273	0.0370	0.0299	0.0240
Di Veroli et al. (2013)	0.0251	0.0375	0.0379	0.0492	0.0204
Mazhari et al. (2001)	0.0319	0.0369	0.0324	0.0606	0.0292
Ten Tusscher et al. (2004)	0.0409	0.0451	0.0455	0.0603	0.0384
Zeng et al. (1995)	0.0488	0.0644	0.0472	0.0688	0.0342

Table D8: Table quantifying mean square difference (units nA) between experimental current traces and simulation predictions for the validation protocols shown in Figures 5 and 6 for Cell #7. Here the color scale is set so that [yellow] represents zero error and [red] represents the highest error for each protocol/column.

Model	Sine Wave (Pr7)	AP (Pr6)	Steady Act. (Pr3)	Deact. (Pr5)	Inact. (Pr4)
New model for Cell #7	0.0495	0.0777	0.1446	0.1824	0.0655
Wang et al. (1997)	0.1175	0.1394	0.1743	0.1392	0.0923
Di Veroli et al. (2013)	0.1142	0.1541	0.2052	0.2382	0.0960
Mazhari et al. (2001)	0.1485	0.1628	0.1410	0.2669	0.1303
Ten Tusscher et al. (2004)	0.1900	0.2102	0.2237	0.2726	0.1700
Zeng et al. (1995)	0.2121	0.2567	0.2567	0.3177	0.1624

Table D9: Table quantifying mean square difference (units nA) between experimental current traces and simulation predictions for the validation protocols shown in Figures 5 and 6 for Cell #8. Here the color scale is set so that [yellow] represents zero error and [red] represents the highest error for each protocol/column.

Model	Sine Wave (Pr7)	AP (Pr6)	Steady Act. (Pr3)	Deact. (Pr5)	Inact. (Pr4)
New model for Cell #8	0.0294	0.0482	0.0551	0.0927	0.0510
Wang et al. (1997)	0.0605	0.0625	0.1112	0.1258	0.0626
Di Veroli et al. (2013)	0.0542	0.0753	0.0858	0.1415	0.0641
Mazhari et al. (2001)	0.0700	0.0759	0.0893	0.1717	0.0831
Ten Tusscher et al. (2004)	0.0864	0.0940	0.1279	0.1810	0.1013
Zeng et al. (1995)	0.0990	0.1168	0.1324	0.1909	0.0961

Table D10: Table quantifying mean square difference (units nA) between experimental current traces and simulation predictions for the validation protocols shown in Figures 5 and 6 for Cell #9. Here the color scale is set so that [yellow] represents zero error and [red] represents the highest error for each protocol/column.

Model	Sine Wave (Pr7)	AP (Pr6)	Steady Act. (Pr3)	Deact. (Pr5)	Inact. (Pr4)
New model for Cell #9	0.0183	0.0314	0.0361	0.0506	0.0294
Wang et al. (1997)	0.0300	0.0351	0.0558	0.0483	0.0339
Di Veroli et al. (2013)	0.0248	0.0307	0.0374	0.0531	0.0277
Mazhari et al. (2001)	0.0262	0.0329	0.0407	0.0632	0.0315
Ten Tusscher et al. (2004)	0.0358	0.0421	0.0612	0.0673	0.0408
Zeng et al. (1995)	0.0429	0.0470	0.0602	0.0709	0.0364

E Additional Current-Voltage Relationship Predictions

Here we show the remainder of the predictions of the current-voltage relationships for the validation data of cell 5 that were not included in the main text (the results of Pr1 and Pr2, and extra IV curves for Pr4). Figure E8 shows the summary curves for Pr1 (voltage clamp shown in Figure B3) and Pr2 (voltage clamp shown in Figure B4).

Traditionally these peak current curves would be plotted by normalizing to the peak current recorded in each activation kinetics protocol. However, as we have used a shorter version of the activation kinetics protocol, we do not expect that the channel would be fully open at the longest duration test step in Pr1 and Pr2. We have therefore instead normalized the curves using the peak current during the initial deactivation step in the sine wave protocol (around 1.6 seconds) where we expect the channel to be maximally open.

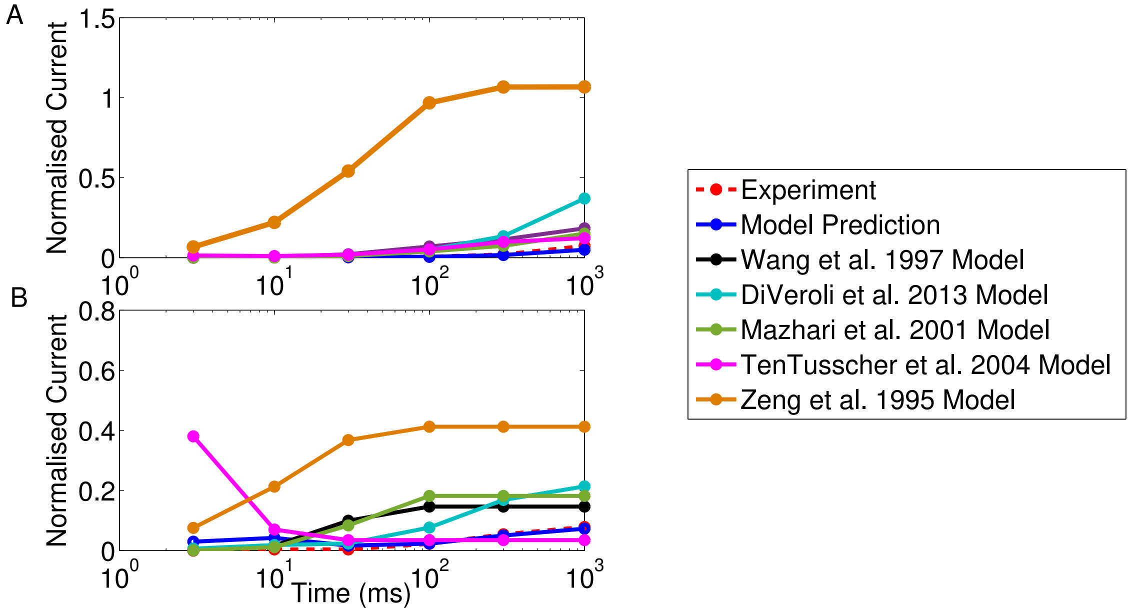

Figure E8: Predictions of peak current-voltage relationship derived from experiment and model predictions in response to; A) Activation Kinetics Pr1, B) Activation Kinetics Pr2, with comparison of our model prediction with predictions from existing literature models. Currents have been normalized to the peak current in the initial deactivation step in the sine wave protocol (around 1.6 seconds) as we do not expect the channel to be fully open at the longest T_{step} in these activation kinetics protocols.

In Figures E9 and E10 we plot additional IV curves summarising the experimental, New Model and literature model responses to the inactivation protocol Pr4.

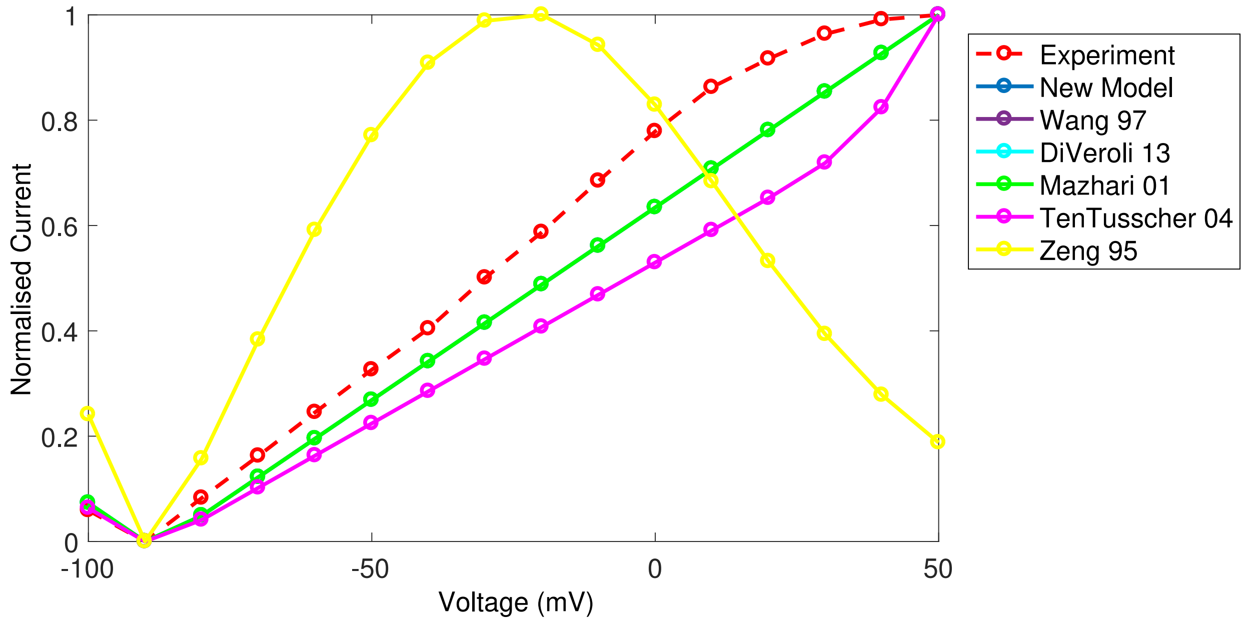

Figure E9: Inactivation peak current IV curve, summarizing currents in response to Pr4. The New Model, Wang, DiVeroli and Mazhari model IV curves are all indistinguishable here and lie on top of one another under the green line.

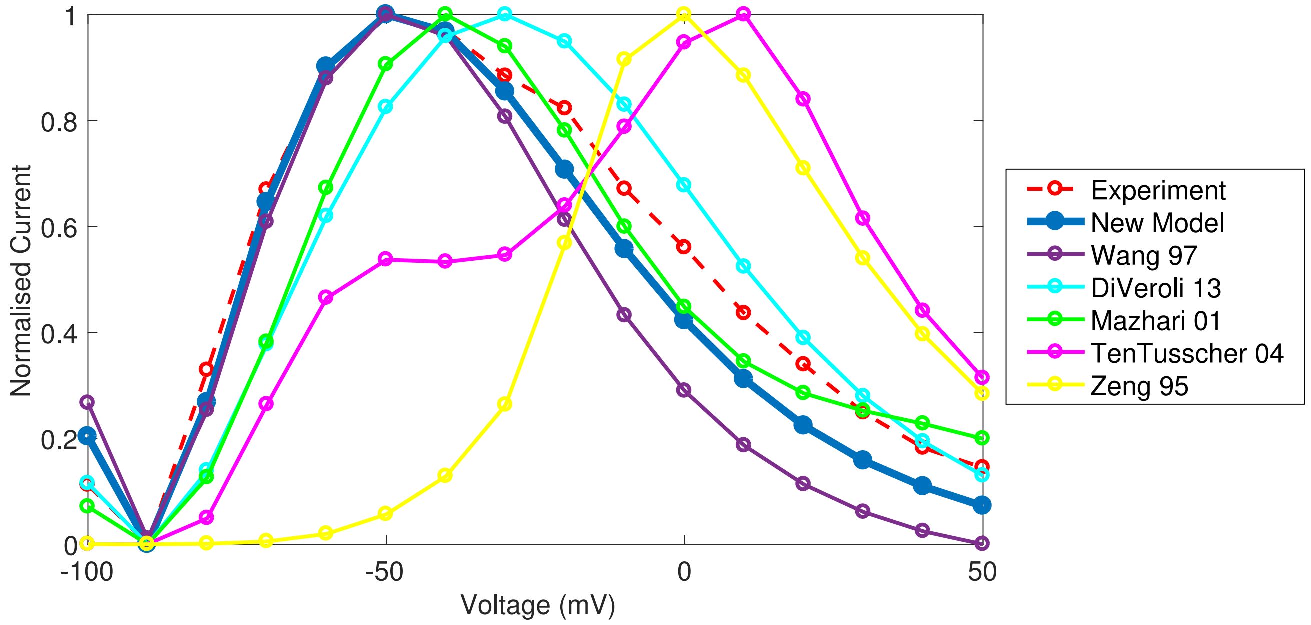

Figure E10: Inactivation steady-state current IV curve, summarizing currents in response to Pr4.

F Comparing Cell-Specific with Average Model

In addition to creating cell specific models as described so far we also created an averaged model by first normalizing each experimental trace to one reference trace (so that each trace was given equal weight in the averaging regardless of the conductance of the channel) and then summing and averaging the current value at each time point along the protocol.

The parameter values obtained when calibrating each cell-specific and averaged model are shown in Table F11. These values correspond to the parameter sets with maximum posterior density identified in the MCMC chain. The full posterior density distributions for each parameter for each of the 9 cells are shown in Figure F11.

Table F11: Table of parameter values at the maximum posterior density for each cell-specific model, and the model fitted to averaged data (N.B. not the average of the cell-specific parameters). Here the model parameter numberings correspond to those detailed in Figure 4B, and G_{Kr} represents the conductance value fitted for each model. *Note that the conductance fitted for the 'Averaged' model reflects mainly the conductance for the reference experimental trace (used for scaling all other traces before averaging), and should not be considered the 'average' conductance, hence its omission from Figure 7A.

	P_1	P_2	P_3	P_4	P_5	P_6	P_7	P_8	G_{Kr}
Cell #1	1.9800×10^{-4}	0.0593	7.1688×10^{-5}	0.0493	0.1048	0.0139	0.0038	0.0360	0.1351
Cell #2	3.2387×10^{-4}	0.0653	7.8195×10^{-5}	0.0497	0.0805	0.0025	0.0049	0.0324	0.0902
Cell #3	4.7771×10^{-4}	0.0661	5.1611×10^{-5}	0.0523	0.1375	0.0094	0.0039	0.0375	0.1011
Cell #4	6.7414×10^{-4}	0.0577	5.8027×10^{-5}	0.0517	0.0893	0.0057	0.0059	0.0324	0.0741
Cell #5	2.2603×10^{-4}	0.0699	3.4481×10^{-5}	0.0546	0.0873	0.0089	0.0052	0.0316	0.1524
Cell #6	6.1840×10^{-4}	0.0658	1.2754×10^{-4}	0.0379	0.0810	0.0165	0.0092	0.0253	0.0218
Cell #7	5.4045×10^{-4}	0.0484	6.5855×10^{-5}	0.0457	0.0627	0.0087	0.0054	0.0318	0.1553
Cell #8	3.1336×10^{-4}	0.0481	5.0647×10^{-5}	0.0491	0.0723	0.0063	0.0060	0.0328	0.0984
Cell #9	5.6194×10^{-4}	0.0433	1.2400×10^{-4}	0.0444	0.0659	0.0028	0.0036	0.0343	0.0514
Averaged	4.0000×10^{-4}	0.0579	6.5092×10^{-5}	0.0487	0.0807	0.0068	0.0052	0.0334	0.0673*

To quantitatively compare the average model predictions and the cell-specific model predictions shown in Figure 7B of the main text we calculated the mean square difference at each point between the average model and the cell-specific models for each cell when predicting the full current trace in response to the steady-state activation protocol. We also repeated this for the deactivation and inactivation protocols and the action potential protocol shown in Figures 5 and 6. The differences for each cell are shown in Table F12 with a comparison between the experimental result and the average model predictions with the cell-specific predictions.

We note that we have ordered the cells in this table (as in Figure 7) according to the percentage change in leak resistance between performing the vehicle and dofetilide repeats of the sine wave voltage protocol used to construct the model. This ordering acts as an estimated ranking for the quality of each recording. The benefit of a cell-specific approach occurs when using the highest quality data for both model construction and validation. We should note that even though in cells #4 and #6 the average model provides the better prediction of the steady-state activation peak current-voltage relationship than the cell-specific model, the cell-specific models are still providing very good predictions in these cases, it is just that the experimental behavior is more like the average model behavior for these cells. We also note that for eight out of the nine cells, the cell-specific model provides a better prediction of the current response to the action potential protocol than the average model, however, in the case where the cell-specific model is worse the difference is only a small amount.

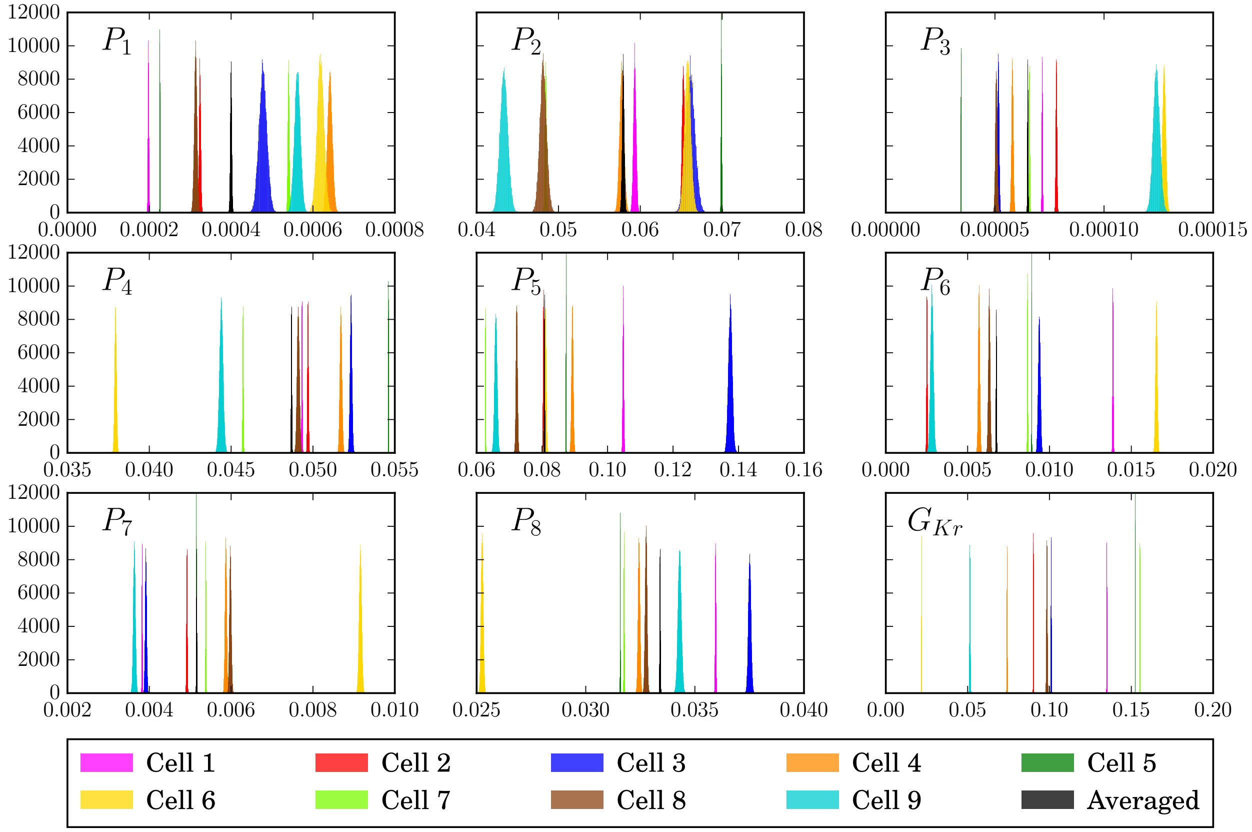

Figure F11: Distributions for each parameter for each of the 9 cell-specific models and the averaged data model. To aid comparison these are all histograms with 100 bars (plotting probability distributions here leads to very different maxima, obscuring the spread information), and so the y-axis is in arbitrary units related to the number of samples. We see that the parameter values tend to be given distinct distributions and so we would consider most of them to be 'significantly different', indicating that the variation we see in Figure 7 is due to cell-cell variability in the recordings rather than noise or unidentifiability in our parameter estimates.

We use a measure of

$$\text{error} = \frac{1}{T}\sum_{t=1}^{T}\Big((\text{simulated current at time step } t - \text{experimental current at time step } t)^2\Big)^{\frac{1}{2}}, \tag{F.10}$$

to evaluate the error in model predictions for individual cells (using the whole current traces, apart from removing regions in the sine wave protocol with capacitive spikes as explained in Methods B2.1).

Table F12: Table showing the error measure defined by equation F.10 between cell-specific or average models and the experimental current recording for fit (sine wave Pr7) and predictions with validation protocols (all other columns). Cells are ordered in ascending order according to the percentage change in leak resistance R_{leak}. Here the color scale is set so that within each pair of columns represents lowest error and represents the highest error for each protocol/pair of columns. Note that the cells with larger currents will show larger errors, but the left column cell-specific predictions tend to perform better than the average model, particularly for cells where the average model gives a relatively large error.

Cell	ΔR_{leak}	Sine Wave (Pr 7)		APs (Pr 6)		Steady Act. (Pr 3)		Deact. (Pr 4)		Inact. (Pr 5)	
#	(%)	Specific	Average	Spec.	Aver.	Spec.	Aver.	Spec.	Aver.	Spec.	Aver.
1	0.0	0.0151	0.0367	0.0283	0.0567	0.0332	0.0770	0.0925	0.1138	0.0312	0.0372
2	7.7	0.0164	0.0271	0.0295	0.0343	0.0270	0.0334	0.0656	0.0619	0.0219	0.0237
3	12.5	0.0253	0.0406	0.0415	0.0681	0.0658	0.1111	0.0785	0.0869	0.0537	0.0731
4	16.7	0.0258	0.0318	0.0359	0.0411	0.0701	0.0580	0.0878	0.0846	0.0522	0.0563
5	20.0	0.0203	0.0228	0.0453	0.0535	0.0437	0.0591	0.1317	0.1608	0.0700	0.0668
6	28.6	0.0113	0.0205	0.0216	0.0250	0.0264	0.0206	0.0504	0.0435	0.0170	0.0183
7	32.5	0.0495	0.0631	0.0777	0.0885	0.1446	0.1198	0.1824	0.2016	0.0655	0.0740
8	42.9	0.0294	0.0352	0.0482	0.0507	0.0551	0.0603	0.0927	0.1265	0.0510	0.0526
9	58.3	0.0183	0.0195	0.0314	0.0306	0.0361	0.0321	0.0506	0.0462	0.0294	0.0294

For predictions of the action potential protocol currents, Table F12 demonstrates that the cell-specific modeling approach yields predictions that are very close to or better than the average model. Additionally, for the predictions of the steady-state activation protocol the cell-specific approach generally yields very good and more accurate (for 4/5) predictions of validation data when the highest quality data is used (cells #1–5). This benefit is absent when lower quality experimental data is used where the average model provides very similar, but slightly better, predictions (cells #6–9).

We also compare cell-specific and average predictions for each of the 9 cells for the deactivation, recovery from inactivation and instantaneous inactivation time constants as were shown for one cell in Figure 5. We show this comparison for each cell in Figure F12 and F13 for 8/9 cells and in Figure F14 for all cells. Cell #6 was omitted in the first two plots because this cell had a particularly low current and it was difficult to accurately fit exponential curves to the experimental data for this cell. We also note that we have not plotted the time constant values for $-90\,\text{mV}$ in Figures F12 & F13 for the same reason; we could not confidently fit an exponential decay curve to determine an accurate time constant value for this voltage step.

We see in Figures F12–F14 that the same observations that were made for the results shown in Figure 7 generally hold: for lower cell numbers #1–5, we see enhanced predictions of the experimental time constants from the cell-specific model rather than the averaged model. i.e predictions are better in the cells with lower percentage changes in leak current resistance, which correspond

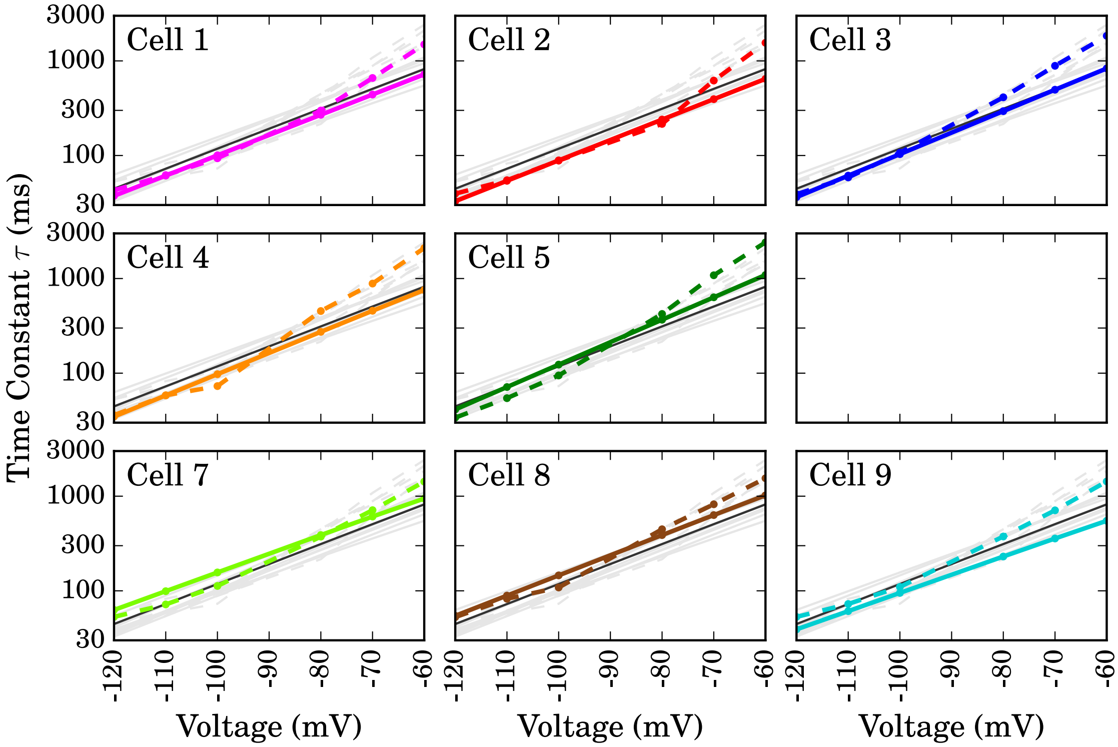

Figure F12: Cell-specific model predictions of time constant/voltage relationships for deactivation (Pr5). Each plot represents a different cell, with cell-specific model prediction depicted by the bold line, and the dashed line showing the cell's experimental data. Black lines on each plot represents the average model prediction. Cells are ordered as in Table F12.

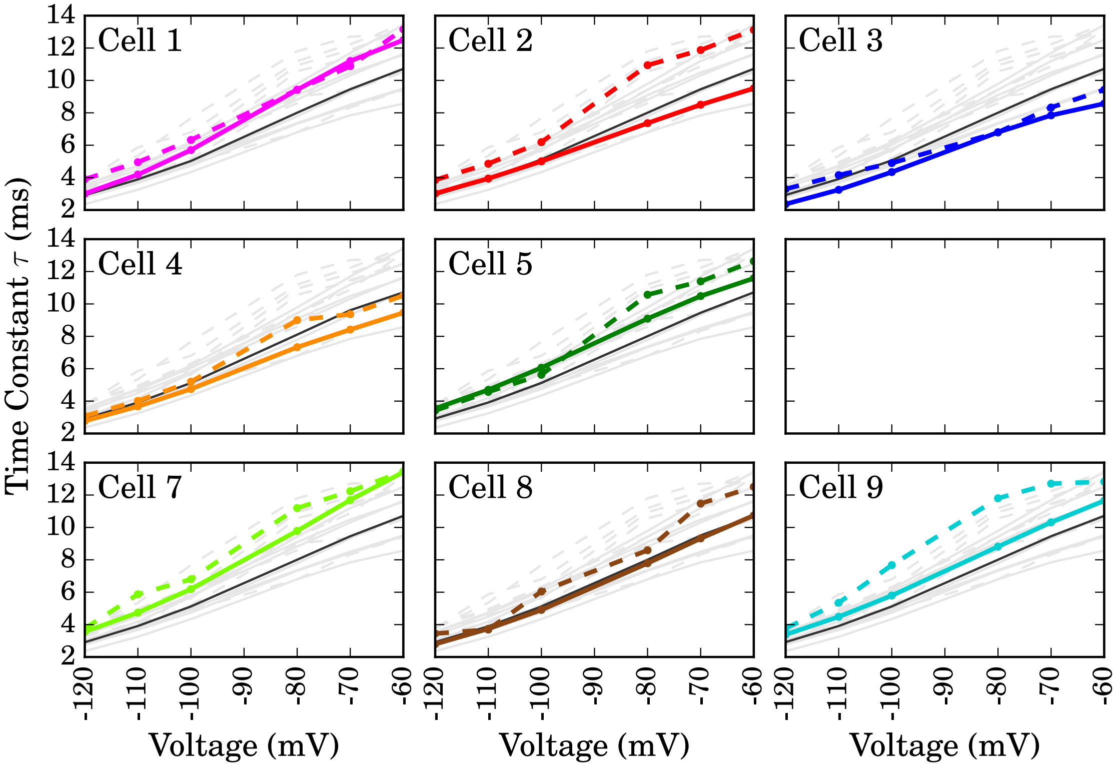

Figure F13: Cell-specific model predictions of time constant/voltage relationships for recovery from inactivation in Pr5. Each plot represents a different cell, with cell-specific model prediction depicted by the bold line, and the dashed line showing the cell's experimental data. Black lines on each plot represents the average model prediction. Cells are ordered as in Table F12.

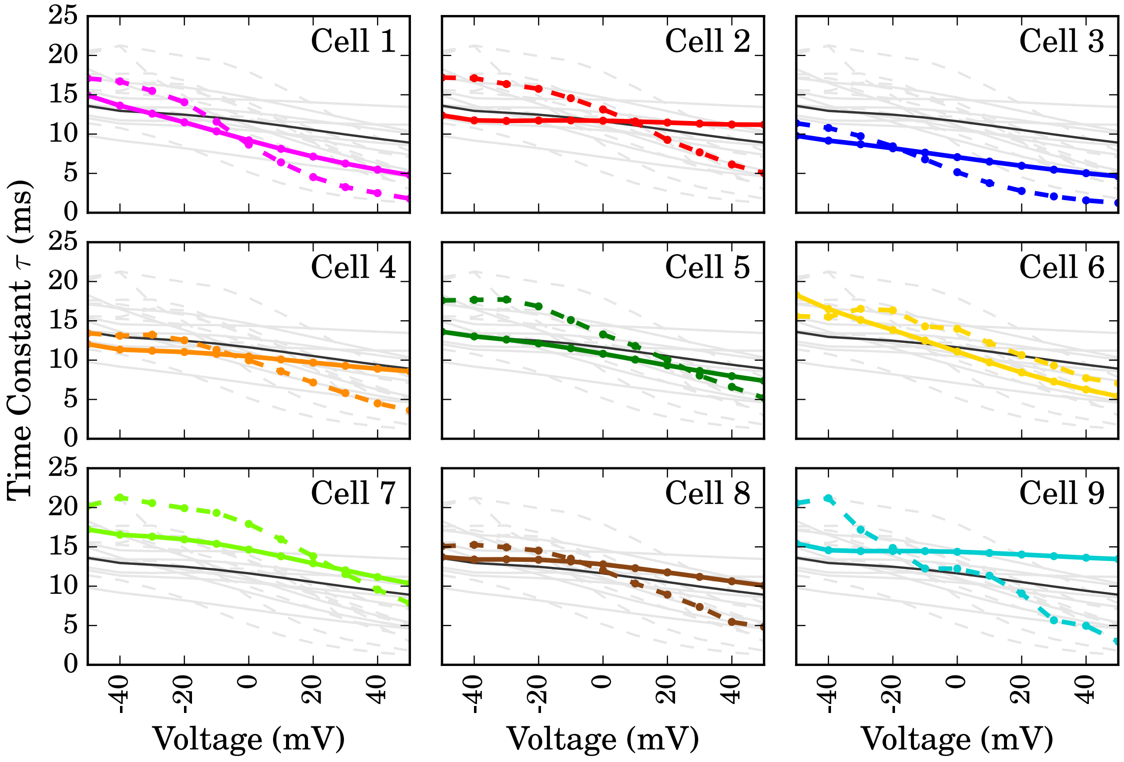

Figure F14: Cell-specific model predictions of time constant/voltage relationships for instantaneous inactivation (Pr4). Each plot represents a different cell, with cell-specific model prediction depicted by the bold line, and the dashed line showing the cell's experimental data. Black lines on each plot represents the average model prediction. Cells are ordered as in Table F12.

to better quality data.

G Synthetic data study for an $\mathrm{I_{Ks}}$ model

In order to demonstrate the wider applicability of our approach we also show its suitability for parameterising a model of a different ion current — the slowly activating delayed rectifier potassium channel ($\mathrm{I_{Ks}}$). We tested the approach using a synthetic data study similar to that described in Section C2 for the model in the main text. The $\mathrm{I_{Ks}}$ model we used was taken from the Ten Tusscher et al. (2004) cardiac action potential model which (including a conductance parameter) has 8 parameters within the $\mathrm{I_{Ks}}$ model. The model, which we have rewritten to make all parameters *a priori* identifiable, takes the form shown in Equations (G.11)–(G.16):

$$I_{Ks} = G_{Ks} X_s^2 (V - E_{Ks}), \tag{G.11}$$

$$Xs_{\infty} = \frac{1}{1 + P_1 \exp(-P_2 V)}, \tag{G.12}$$

$$\alpha_{Xs} = \frac{1}{1 + P_6 \exp(P_7 V)}, \tag{G.13}$$

$$\beta_{Xs} = \frac{1}{P_5 \sqrt{1 + P_3 \exp(-P_4 V)}}, \tag{G.14}$$

$$\tau_{Xs} = \alpha_{Xs} \beta_{Xs}, \tag{G.15}$$

$$\frac{\mathrm{d}X_s}{\mathrm{d}t} = \frac{Xs_{\infty} - X_s}{\tau_{Xs}}. \tag{G.16}$$

In Table G13 we compare the parameters we used to generate the synthetic data and the parameters we identified as producing the best fit to these synthetic data (which included added noise). We see that the maximum likelihood parameters are very similar to those from which the synthetic data were produced, demonstrating the theoretical capability of this approach to parameterise a model of $\mathrm{I_{Ks}}$. The practicality of this approach with real data is still to be explored.

Table G13: Table comparing parameter values used to simulate the synthetic data for the $\mathrm{I_{Ks}}$ model and the maximum likelihood fit to these data.

	P_1	P_2	P_3	P_4	P_5	P_6	P_7	G_{Kr}
Underlying Synthetic Data Value	0.6997	0.0714	0.1889	0.1667	0.0009	0.0498	0.05	0.1
Fitted Value	0.6967	0.0716	0.1964	0.1659	0.0009	0.0540	0.0486	0.0999

H Testing with a five state Markov model

In this section we test whether the sinusoidal protocol is capable of fitting a more complicated model of hERG kinetics with both more states and more parameters. We consider the five state model structure of Wang et al. (1997), as shown in Figure H15B. This model has 14 kinetic parameters, rather than the 8 in our Hodgkin-Huxley style model, we maintain the non-voltage-dependent transition rates between states C_2 and C_1 (states and parameters labelled as depicted in Figure H15B) which was proposed by Wang et al. (1997), which was suggested as the most likely hERG model in Bett et al. (2011). Figure H15A shows the fit to the sinusoidal protocol Pr6 for Cell #5 data (analogous to Figure 4 of the main text).

We see a very good fit to the training protocol with almost all parameters having narrow posterior distributions, as we did with the Hodgkin-Huxley (HH) model. The mean square difference in the fit with this five state model is 0.0164 nA as compared to 0.0203 nA when fitting the HH model (this is no surprise as we expect a model with more parameters to be able to get a closer fit), we can see the main difference is in better fits of the multiple time constants in the response to the 'closing' voltage-step at 1.5–2 s and 6.5–7 s.

The only parameter of particular note here is P_2 (the voltage-dependence of the C_1 to O transition), it's posterior distribution is 'hitting' the lower end of the prior, hinting that there may be no/low voltage dependence on this transition. We surmise that either: this voltage-dependence is not required to fit the sinusoidal protocol — there is insufficient information on this parameter revealed by the protocol (although note the posterior is heavily skewed towards zero, not just keeping the same shape as the prior, suggesting there is information and P_2 needs to be small); or the channel really does have little voltage dependence here — perhaps even that the diagram could have a closed state removed and the channel function can be equally well represented with a simpler model.

In Figure H16 we show the predictions of the parameterised five state model for the action potential protocol Pr6, akin to that shown in Figure 6 in the main text. We see that the five state model is quite predictive for this protocol, and has much less error than with its original parameters (shown in purple in panel E and quantified as 0.0764 nA in Table D6 above). Across the whole of Protocol 6 the mean square error for this five state model is slightly less than it was for the new HH model (0.0419 nA vs. 0.0453 nA respectively). We attribute this difference to the better fit of the multiple time constants in the channel closing after 0.73 s, because if we consider just the main action potential section (0.5702–7.3245 seconds of Pr6), the mean square error for five state model is slightly more than the model in the main text (0.0475 nA vs. 0.0471 nA respectively).

The larger structure also makes slightly larger errors when predicting the standard voltage-step protocols, shown in Figure H17 below. We draw attention to the activation, inactivation and recovery from inactivation summary curves, all of which are slightly worse under the five-state model than under the HH model (see Figure 5 of the main text).

Overall, despite an improvement in model fit (comparing Figure H15 in this response with Figure 4 in the main text), we see a slight deterioration in model predictions (a common pitfall when complicating a model, known as "variance bias trade-off" in statistical models), perhaps suggesting we are on the boundary of 'over-fitting' in terms of either the number parameters or complexity of the model structure. This finding emphasises the value of a simple model to optimise accuracy in model parameterisation and predictive power. In parallel work, we are exploring and extending the use of this methodology for both parameterising *and selecting* the most representative and predictive model of hERG channel kinetics. The findings here suggest that perhaps a compromise between a structure capable of multiple time constants in closing whilst retaining simplicity may be the most appropriate choice.

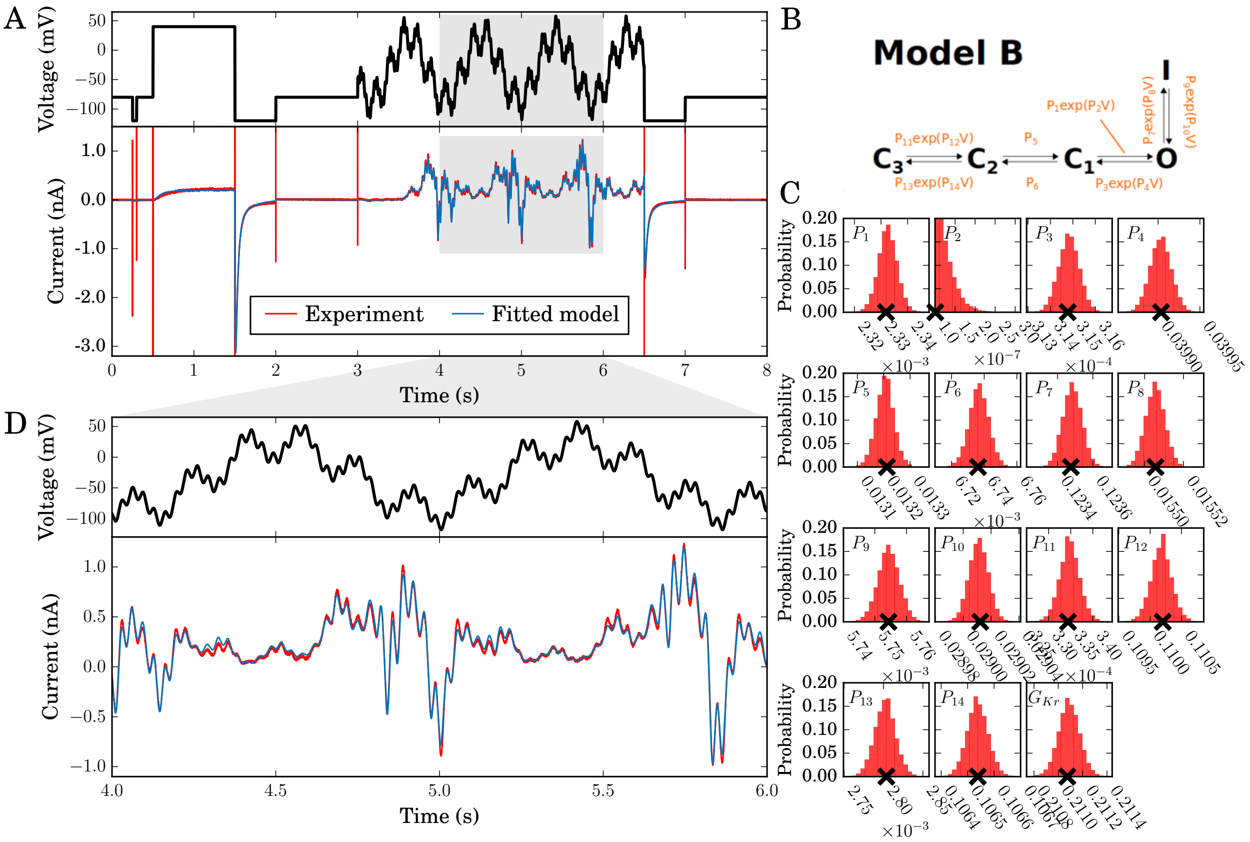

Figure H15: **Model calibration for the five-state model**. For comparison with Figure 4 of the main text. **A:** Top: training protocol, bottom: an experimental recording with the fitted model simulation overlaid. This simulation uses the maximum posterior density parameter set, denoted with crosses in panel C. **B:** The five state model structure in Markov state diagram format. Parameter values P_1 to P_{14} define transitions between conformational states. The transition between C_2 and C_1 states are assumed to be voltage independent, as in Wang et al. (1997). **C and D:** as per main text Figure 4.

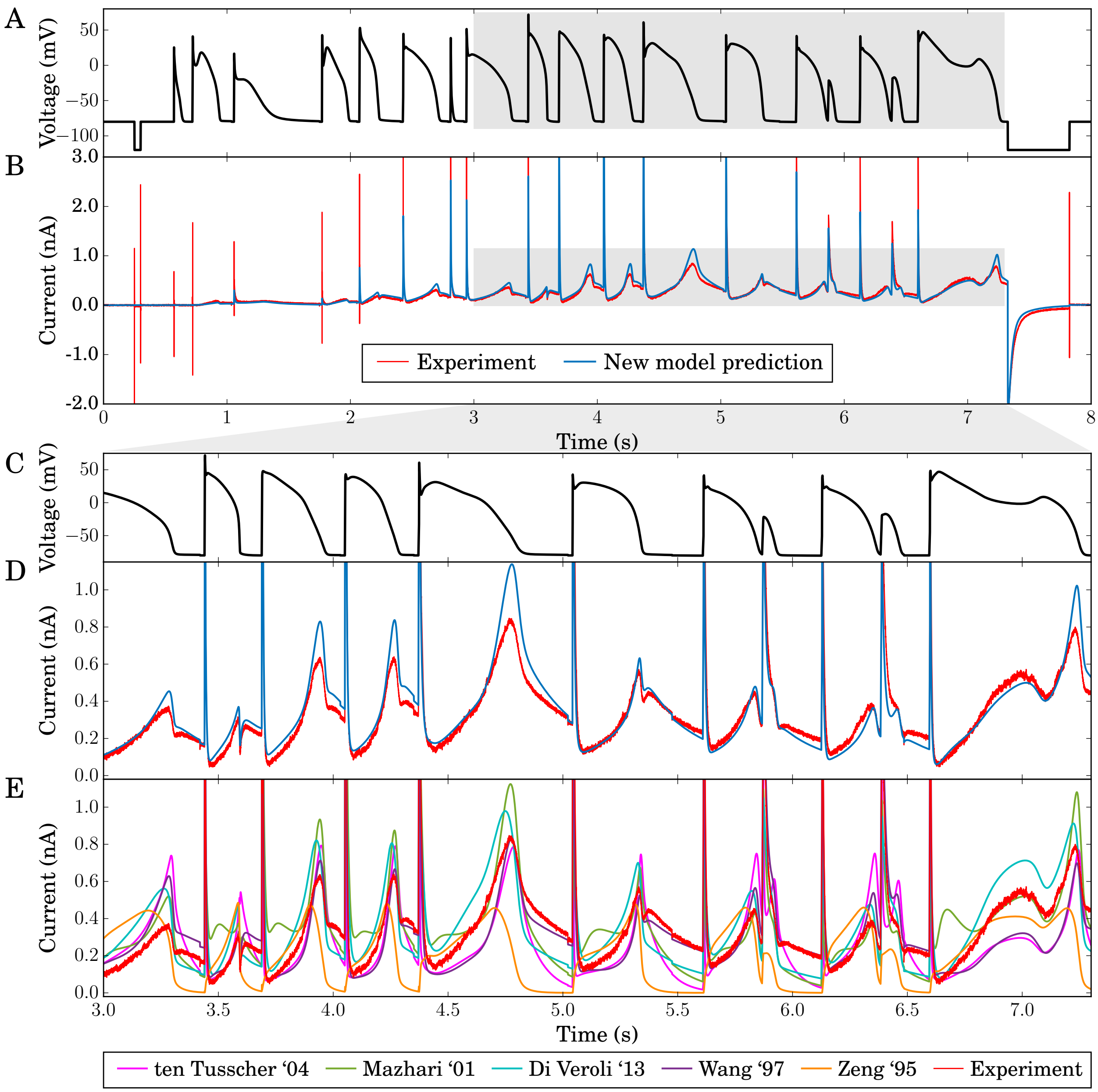

Figure H16: **Validation prediction for the five-state model** — the current in response to the action potential protocol, for comparison with Figure 6 of the main text.

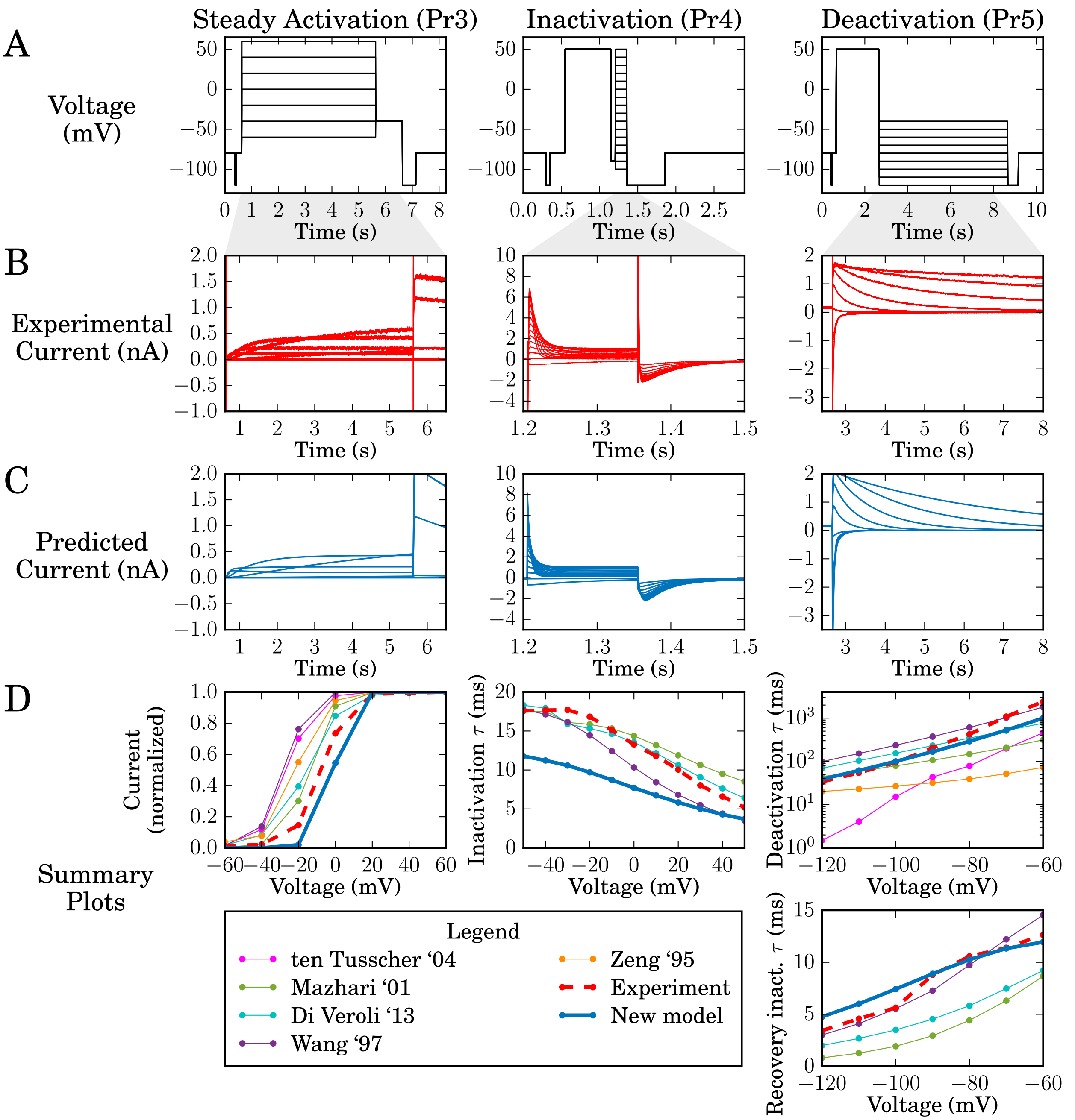

Figure H17: **Validation predictions for the five-state model** — currents in response to traditional voltage step protocols, for comparison with Figure 5 of the main text.

References

Aslanidi OV, Stewart P, Boyett MR & Zhang H (2009). Optimal velocity and safety of discontinuous conduction through the heterogeneous purkinje-ventricular junction. *Biophysical Journal* **97**, 20–39.

Bett GC, Zhou Q & Rasmusson RL (2011). Models of HERG Gating. *Biophysical Journal* **101**, 631–642.

Clancy C & Rudy Y (2001). Cellular consequences of HERG mutations in the long QT syndrome: Precursors to sudden cardiac death. *Cardiovascular Research* **50**, 301–313.

Courtemanche M, Ramirez R & Nattel S (1998). Ionic mechanisms underlying human atrial action potential properties: Insights from a mathematical model. *American Journal of Physiology-Heart and Circulatory Physiology* **275**, H301–H321.

Di Veroli G, Davies M, Zhang H, Abi-Gerges N & Boyett M (2013). High-throughput screening of drug-binding dynamics to HERG improves early drug safety assessment. *American Journal of Physiology-Heart and Circulatory Physiology* **304**, H104–H117.

Fink M, Noble D, Virag L, Varro A & Giles WR (2008). Contributions of HERG K+ current to repolarization of the human ventricular action potential. *Progress in Biophysics and Molecular Biology* **96**, 357–376.

Fox JJ, McHarg JL & Gilmour Jr RF (2002). Ionic mechanism of electrical alternans. *American Journal of Physiology-Heart and Circulatory Physiology* **282**, H516–H530.

Gilks WR, Richardson S & Spiegelhalter DJ (1996). *Markov Chain Monte Carlo in Practice* London: Chapman and Hall.

Grandi E, Pasqualini FS & Bers DM (2010). A novel computational model of the human ventricular action potential and Ca transient. *Journal of Molecular and Cellular Cardiology* **48**, 112–121.

Haario H, Saksman E & Tamminen J (2001). An Adaptive Metropolis Algorithm. *Bernoulli* pp. 223–242.

Hund TJ & Rudy Y (2004). Rate dependence and regulation of action potential and calcium transient in a canine cardiac ventricular cell model. *Circulation* **110**, 3168–3174.

Inada S, Hancox J, Zhang H & Boyett M (2009). One-dimensional mathematical model of the atrioventricular node including atrio-nodal, nodal, and nodal-his cells. *Biophysical Journal* **97**, 2117–2127.

Kiehn J, Lacerda A & Brown A (1999). Pathways of HERG inactivation. *American Journal of Physiology-Heart and Circulatory Physiology* **277**, H199–H210.

Kurata Y, Hisatome I, Imanishi S & Shibamoto T (2002). Dynamical description of sinoatrial node pacemaking: improved mathematical model for primary pacemaker cell. *American Journal of Physiology-Heart and Circulatory Physiology* **283**, H2074–H2101.

Lacroix JJ, Labro AJ & Bezanilla F (2011). Properties of deactivation gating currents in shaker channels. *Biophysical Journal* **100**, L28–L30.

Lindblad D, Murphey C, Clark J & Giles W (1996). A model of the action potential and underlying membrane currents in a rabbit atrial cell. *American Journal of Physiology* **271**, H1666–H1696.

Liu S, Rasmusson R, Campbell D, Wang S & Strauss H (1996). Activation and inactivation kinetics of an E-4031-sensitive current from single ferret atrial myocytes. *Biophysical Journal* **70**, 2704–2715.

Lu Y, Mahaut-Smith M, Varghese A, Huang C, Kemp P & Vandenberg J (2001). Effects of premature stimulation on HERG K+ channels. *The Journal of Physiology* **537**, 843–851.

Matsuoka S, Sarai N, Kuratomi S, Ono K & Noma A (2003). Role of individual ionic current systems in ventricular cells hypothesized by a model study. *The Japanese Journal of Physiology* **53**, 105–123.

Mazhari R, Greenstein J, Winslow R, Marbán E & Nuss H (2001). Molecular interactions between two Long-QT syndrome gene products, HERG and KCNE2, rationalized by in vitro and in silico analysis. *Circulation Research* **89**, 33–38.

Niederer S, Fink M, Noble D & Smith N (2009). A meta-analysis of cardiac electrophysiology computational models. *Experimental Physiology* **94**, 486–495.

Nygren A, Fiset C, Firek L, Clark J, Lindblad D, Clark R & Giles W (1998). Mathematical model of an adult human atrial cell the role of K+ currents in repolarization. *Circulation Research* **82**, 63–81.

Oehmen C, Giles W & Demir S (2002). Mathematical model of the rapidly activating delayed rectifier potassium current IKr in rabbit sinoatrial node. *Journal of Cardiovascular Electrophysiology* **13**, 1131–1140.

O'Hara T, Virág L, Varró A & Rudy Y (2011). Simulation of the undiseased human cardiac ventricular action potential: model formulation and experimental validation. *PLoS Computational Biology* **7**, e1002061.

Piper D, Varghese A, Sanguinetti M & Tristani-Firouzi M (2003). Gating currents associated with intramembrane charge displacement in HERG potassium channels. *Proceedings of the National Academy of Sciences* **100**, 10534.

Priebe L & Beuckelmann DJ (1998). Simulation study of cellular electric properties in heart failure. *Circulation Research* **82**, 1206–1223.

Ramirez RJ, Nattel S & Courtemanche M (2000). Mathematical analysis of canine atrial action potentials: rate, regional factors, and electrical remodeling. *American Journal of Physiology-Heart and Circulatory Physiology* **279**, H1767–H1785.

Seemann G, Sachse FB, WEIß DL & DÖSSEL O (2003). Quantitative reconstruction of cardiac electromechanics in human myocardium. *Journal of Cardiovascular Electrophysiology* **14**, S219–S228.

Severi S, Fantini M, Charawi LA & DiFrancesco D (2012). An updated computational model of rabbit sinoatrial action potential to investigate the mechanisms of heart rate modulation. *The Journal of Physiology* **590**, 4483–4499.

Shannon T, Wang F, Puglisi J, Weber C & Bers D (2004). A mathematical treatment of integrated Ca dynamics within the ventricular myocyte. *Biophysical Journal* **87**, 3351–3371.

Ten Tusscher K, Noble D, Noble P & Panfilov A (2004). A model for human ventricular tissue. *American Journal of Physiology-Heart and Circulatory Physiology* **286**, H1573–H1589.

Vandenberg J, Perry M, Perrin M, Mann S, Ke Y & Hill A (2012). hERG K+ channels: Structure, function, and clinical significance. *Physiological Reviews* **92**, 1393–1478.

Walch OJ & Eisenberg MC (2016). Parameter identifiability and identifiable combinations in generalized hodgkin–huxley models. *Neurocomputing* **199**, 137–143.

Wang S, Liu S, Morales M, Strauss H & Rasmusson R (1997). A quantitative analysis of the activation and inactivation kinetics of HERG expressed in Xenopus oocytes. *Journal of Physiology* **502**, 45–60.

Winslow RL, Rice J, Jafri S, Marban E & O'Rourke B (1999). Mechanisms of altered excitation-contraction coupling in canine tachycardia-induced heart failure, II Model studies. *Circulation Research* **84**, 571–586.

Zeng J, Laurita KR, Rosenbaum DS & Rudy Y (1995). Two components of the delayed rectifier K+ current in ventricular myocytes of the guinea pig type theoretical formulation and their role in repolarization. *Circulation Research* **77**, 140–152.

Zhang H, Holden A, Kodama I, Honjo H, Lei M, Varghese T & Boyett M (2000). Mathematical models of action potentials in the periphery and center of the rabbit sinoatrial node. *American Journal of Physiology-Heart and Circulatory Physiology* **279**, H397–H421.
